# Digitizing Survivorship Care Plans Through the POST-Treatment Health Outcomes of Cancer Survivors (POSTHOC) Mobile App: Protocol for a Phase II Randomized Controlled Trial

**DOI:** 10.2196/59222

**Published:** 2024-09-05

**Authors:** Kaitlin H Chung, Shari M Youngblood, Carin L Clingan, Dana C Deighton, Virginia A Jump, Thushini Manuweera, Nicolette M McGeorge, Cynthia L Renn, Paula Y Rosenblatt, Aaron T Winder, Shijun Zhu, Ian R Kleckner, Amber S Kleckner

**Affiliations:** 1 Department of Pain and Translational Symptom Science University of Maryland School of Nursing Baltimore, MD United States; 2 Cornell University Ithaca, NY United States; 3 Department of Integrative and Functional Nutrition Saybrook University Pasadena, CA United States; 4 University of Maryland Greenebaum Comprehensive Cancer Center Baltimore, MD United States; 5 University of Maryland Medical Center Baltimore, MD United States; 6 St. Joseph Medical Center Towson, MD United States; 7 Charles River Analytics Cambridge, MA United States; 8 University of Maryland School of Medicine Baltimore, MD United States; 9 Department of Organizational Systems and Adult Health University of Maryland School of Nursing Baltimore, MD United States

**Keywords:** mobile health, mHealth, symptoms, clinical trial, posttreatment, oncology, mobile phone

## Abstract

**Background:**

Survivorship care plans (SCPs) are provided at the completion of cancer treatment to aid in the transition from active treatment to long-term survivorship. They describe the details of a patient’s diagnosis and treatment and offer recommendations for follow-up appointments, referrals, and healthy behaviors. The plans are currently paper-based and become outdated as soon as a patient’s health status changes. There is a need to digitize these plans to improve their accessibility, modifiability, and longevity. With current technology, SCPs can be linked to mobile devices and activity trackers so that patients can track health behaviors and compare them to their clinical goals, taking charge of their own health.

**Objective:**

A mobile app, POSTHOC (POST-Treatment Health Outcomes of Cancer Survivors), that digitizes the SCP was developed, with goals of integrating it with wearable technologies and electronic medical records. Herein, we are conducting a randomized controlled trial that evaluates the POSTHOC app versus the traditional SCP on total symptom burden in the early posttreatment period.

**Methods:**

We will recruit 54 patients who have recently completed curative therapy for cancer (any type) in person and remotely. They will be randomized 2:1, POSTHOC:usual care (unblinded). Those randomized to the POSTHOC group will receive their SCP via the app and will choose to focus on nutrition or exercise for the duration of the study based on their individual plan and personal preferences. Those randomized to the control group will get a paper-based plan. At baseline, 6 weeks, and 12 weeks, we will evaluate patient-reported outcomes, including total symptom burden (web-based questionnaire), diet (24-hour Automated Self-Administered [ASA24]), and physical activity (Fitbit Charge 6 [Google LLC]). We will also collect quantitative and qualitative feedback on the usability of the app from those in the POSTHOC arm to improve the app for future implementation studies, with a specific focus on patient-provider communication. For feasibility, we will calculate the percentage of patients who used the POSTHOC app at least 3 times per week. We will use linear mixed models to evaluate the effects of the POSTHOC app versus those of usual care on other outcomes at weeks 6 and 12.

**Results:**

This trial is open to accrual in the University of Maryland Medical System as of March 2024, and as of July 3, 2024, a total of 20 participants have consented.

**Conclusions:**

This study is among the first to digitize the SCP in a mobile app and test the effects of a mobile health–delivered behavioral health intervention on symptom burden in cancer survivors. Our results will provide evidence about the effects of health self-management on symptoms. This knowledge will be integral to larger randomized controlled studies, integration with the electronic medical record, and nationwide implementation.

**Trial Registration:**

ClinicalTrials.gov NCT05499663; https://clinicaltrials.gov/ct2/show/NCT05499663

**International Registered Report Identifier (IRRID):**

DERR1-10.2196/59222

## Introduction

### Background

As of 2022, more than 18 million Americans had a medical history that included cancer, and the prevalence of cancer is projected to continue to increase [1]. This growth has raised concerns about whether the health care industry is adequately prepared to meet the unique health care needs of cancer survivors. Years after treatment ends, cancer survivors continue to cope with symptoms of the disease and side effects of the treatment, including physical limitations, cognitive sequelae, depression, sleep problems, fatigue, and pain. A survey of demographically diverse cancer survivors in California estimates that the average survivor has 5 comorbidities and that 40% of these conditions arise after treatment ends [2]. As cancer rates rise and treatment continues to improve, the size of the cancer survivor population and survivorship duration will continue to increase [3]. This growth trend necessitates the health care industry to develop new tools to support cancer survivors during the posttreatment period to promote long‑term clinical and supportive care outcomes.

The health care community has already taken some actions to address posttreatment challenges. As recommended by the National Academy of Medicine (formally the Institute of Medicine), all cancer survivors should receive a survivorship care plan (SCP) from their oncology clinical care team after their primary treatment ends [4]. The American College of Surgeons Commission on Cancer set SCP guidelines to recommend that survivors receive information regarding future check-ups and tests, education on potential late and long-term effects of the cancer treatment, and recommendations for improving health after treatment [5]. However, current practices for the continued care of survivors are burdensome; are complex; must be continuously updated; and require that survivors, oncologists, and primary care providers coordinate efforts to monitor for recurrence, monitor physical and psychosocial effects of treatment, and promote healthy living [6,7]. Due to this complexity and infrequent communication between survivors and their care team between scheduled check-ups, these practices are not enough to ensure timely symptom identification and adherence to recommendations [8]. Additional tools are needed to supplement the SCP to promote self-management, encourage the achievement or maintenance of healthy lifestyle behaviors, and facilitate the detection of risk for posttreatment complications.

One way to address issues with current SCP practices is through mobile health (mHealth) interventions. mHealth data capabilities have grown significantly due to a greater adoption of smart devices, improved mobile network coverage, and increased focus of American culture on self-monitoring and preventative health [9]. Indeed, interest in mHealth interventions to enhance overall wellness has increased exponentially in the last decade [10], including among patients with cancer [11]. mHealth apps that promote exercise have a variety of functions, including easy visualization of step data from an activity tracker, notifications to promote activity (at set times and in response to sedentary behavior, as detected from participants’ activity tracker) [12], a newsfeed, educational information, easy visualization of users’ activity compared to their goals or prescriptions, peer-support via 2-way messaging with other participants, and symptom tracking [11,13,14]. mHealth apps to deliver nutrition or healthy eating interventions are less prevalent than those that deliver physical activity interventions, although nutrition-related mHealth interventions among cancer survivors have promoted weight loss with preliminary success [15-17]. In addition to enabling the self-monitoring of physical activity and nutrition, mHealth has advantages for researchers in monitoring ambulatory behaviors and soliciting immediate responses to questions, known as ecological momentary assessment (EMA) [18]. Specifically, EMA provides an unprecedented opportunity to capture changes in symptoms over a single day and model how symptoms can predict lifestyle behaviors later that day or how lifestyle behaviors can acutely affect symptoms [19].

To our knowledge, 4 SCP mobile apps are under development [20-24], addressing requests from patients, clinicians, and organizations. First, Baseman et al [20] evaluated the feasibility and acceptability of SmartSurvivor, a mobile breast cancer survivorship care app, in a pilot study. The goals of the app were to facilitate care coordination; support medical, psychosocial, and practical needs; and improve survivors’ long-term health outcomes [20]. Overall, both survivors and providers felt that SmartSurvivor was a potentially valuable tool to support long-term SCP objectives [20]. Second, Denzen et al [22] and Preussler et al [21] are designing a web-based survivorship program for recipients of hematopoietic cell transplants called INSPIRE (Internet Survivorship Program with Information and Resources), which includes a mobile app. In focus groups, patients appreciated the comprehensiveness of the information provided, noting that some material was not provided by their providers [21]. They also noted its usefulness in guiding communication with physicians. However, they would have liked to receive more personalized information (eg, on transplant-induced gastrointestinal upset) as well as a means to monitor and track certain aspects of health [21]. Third, Murphy et al [23] are designing a mobile app to deliver the SCP to survivors of childhood cancer. The authors previously tested a portable, credit card–sized passport to improve the survivors’ knowledge of diagnosis, treatment, risks, and follow-up care. Patients who received the passport demonstrated improved and sustained knowledge compared to those who did not receive the passport until >4 months later [23]. The authors are following up on these results to develop a survivorship mobile phone app and suggest further study on the strengths and weaknesses of a mobile app approach [23]. Fourth, Stan et al [24] are designing an app-based, eHealth record–integrated interactive care plan for breast cancer survivors. The goals of the app are to make the SCP into an actionable and engaging guide for patients and provide remote monitoring services for primary care providers [24]. The implementation of the app with 23 patients was feasible and created minimal provider burden, but patient engagement was below the feasibility threshold, suggesting that changes may enhance adoption [24]. There are other digital applications and mobile apps designed to aid cancer survivors with self-management, but they do not deliver the SCP [25-27].

Qualitative and feasibility studies have concluded that people who have had a cancer diagnosis find both health-tracking apps and SCP useful [10,15,16,20-22,28,29]. However, despite the high number of trials conducted thus far, the apps did not incorporate wearable devices or health tracking, the studies tended to be small and underpowered to detect changes in physical activity patterns, and the projects incorporated only limited feedback from clinicians.

### This Study

This study will fill a significant gap in the literature by being the first study, to our knowledge, to both digitize the SCP in a mobile app integrated with a wearable activity tracker and test the effects of the app on symptom burden in cancer survivors. We hypothesize that (1) the POSTHOC (POST-Treatment Health Outcomes of Cancer Survivors; Charles River Analytics) app is feasible, acceptable, and useful for cancer survivors; (2) the POSTHOC app will reduce cumulative symptom burden reported by patients compared to usual care; and (3) the POSTHOC app will improve health behaviors compared to usual care, as measured by patient-reported physical activity. The results of this study will be built upon to improve the usefulness of the app and integrate it into clinical workflow.

## Methods

### Ethical Considerations

Ethics approval was obtained from the University of Maryland Greenebaum Comprehensive Cancer Center (UMGCCC) Clinical Research Center (GCCC2280), the University of Maryland School of Nursing Office of Research and Scholarship (HP-00100473), the University of Maryland Baltimore (UMB) Institutional Review Board (HP-00100473), and 4 community oncology practices in the University of Maryland Medical System. This study was registered at ClinicalTrials.gov (NCT05499663). All participants will provide informed consent (see Multimedia Appendices 1 and 2 for more details). Participants are compensated $50 USD for their time to participate in the study and may keep the Fitbit activity tracker.

Because the SCP includes protected health information, we are ensuring the safety of the data using several layers of security. First, the SCP includes minimal protected health information that could be used in a security breach; it does not include name, birthday, address, phone number, email address, or medical record number. Second, the SparkCare platform, which houses the POSTHOC app and the PostgreSQL database, is installed on UMB servers managed using Azure (Microsoft Corp) services, so data are not transferred to or from Charles River Analytics or anywhere other than the participants’ personal smartphones. Third, we installed an Internet Information Services (Microsoft Corp) webserver; obtained a public IP address, domain name, and security certificate; and configured a domain name system. The POSTHOC app is not publicly available and can be accessed only via an internal developer link that can be opened only via an invitation (via Test Flight for Apple users).

The POSTHOC app also has a “clinician portal” on a dual authentication–protected website. The clinician portal allows study team members to invite participants, manage participants, assign participants to the POSTHOC or usual care arm, and enter the SCP. Only the study team has access to the study data, although participants may share the data with their provider at their discretion.

### Aims, Design, and Setting

#### Aims

The primary aim is to assess the feasibility, acceptability, and usability of the POSTHOC app among cancer survivors (randomized to the app group: 36/54, 67%). The secondary aim is to obtain a preliminary estimate of the effect of the POSTHOC app versus usual care on cumulative patient-reported symptom burden (approximately 36/54, 67% in the intervention arm and approximately 18/54, 33% in the usual care arm). The tertiary aim is to obtain a preliminary estimate of the effect of the POSTHOC app versus usual care on patient-reported and objectively measured health behaviors (physical activity, diet, and sleep). Exploratory mechanistic aims are to (1) assess cross-sectional associations between health behaviors (physical activity, diet, and sleep) and cumulative symptom burden at baseline; (2) explore associations between changes in health behaviors (physical activity, diet, and sleep) and cumulative symptom burden from baseline to 6 weeks and from baseline to 12 weeks; and (3) explore associations between adherence to the intervention and changes in the same measures—symptoms, physical activity, diet, and sleep.

#### Design and Setting of the Study

This study is a phase II feasibility and preliminary efficacy, parallel-arm, randomized controlled trial conducted via the University of Maryland School of Nursing. Recruitment will occur at the University of Maryland Medical System cancer centers, including the UMGCCC in Baltimore, Maryland; the Cancer Center at University of Maryland (UM) Shore Regional Health in Easton, Maryland; the Cancer Institute at UM St. Joseph Medical Center in Towson, Maryland; the Kaufman Cancer Center at UM Upper Chesapeake Health in Bel Air, Maryland; and the Tate Cancer Center at UM Baltimore Washington Medical Center in Glen Burnie, Maryland. We will recruit 54 cancer survivors at an expected recruitment rate of 3 to 5 participants per month for 12 to 18 months.

### Study Procedures

#### Recruitment, Screening, and Baseline Data Collection

Potential participants will be identified using four methods: (1) screening medical records, (2) direct referral from providers (eg, nurses, nurse navigators, and physicians), (3) flyers, and (4) advertising via standard UMB networks (eg, ResearchMatch). After a potential participant has shown an initial interest in the study, the study team will review eligibility and send the consent form, which includes a detailed discussion of the study activities (see Multimedia Appendix 1 for a copy of the consent form). If the person volunteers to participate, study personnel can obtain informed consent in person or remotely via phone or videoconference and electronic remote consent.

To be eligible for the study, participants must (1) be aged ≥18 years; (2) have had a cancer diagnosis (any type); (3) soon complete treatment or have recently completed treatment (within the past 12 weeks) with chemotherapy, radiotherapy, or surgery with curative intent; (4) have received, plan to receive, or be open to receiving an SCP as per their provider; (5) have access to a device capable of running the POSTHOC app and Fitbit [Google LLC] app (eg, Android [Google LLC] or Apple [Apple, Inc] smartphone) and reliable internet access; (6) be able to read and understand English; and (7) be able to provide written informed consent. Participants must not have planned surgery, radiotherapy, or chemotherapy during the study period (hormonal and biological therapy is allowed). Digital literacy is an implicit *de facto* eligibility criterion.

Participation is voluntary, and all participants are free to withdraw from the study at any time for any reason. For example, if someone becomes ineligible for the study after they consent or if they have recurrence or a surgery planned while they are on study, they will not be withdrawn from the study unless their clinical care team feels that withdrawal is the most appropriate decision.

**Figure 1:**
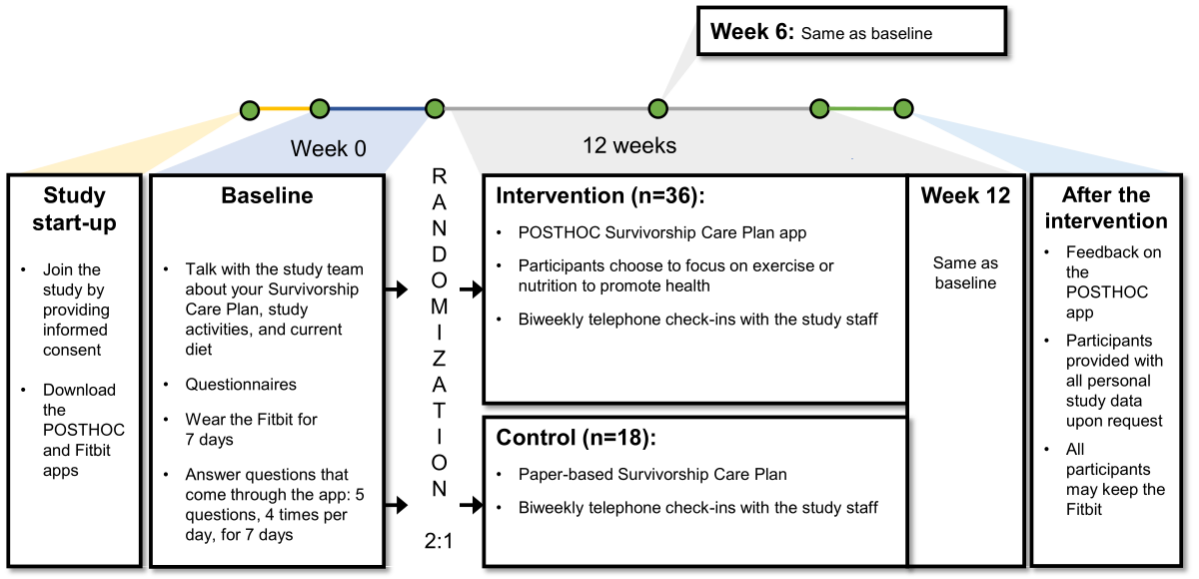
Study schema. In this randomized controlled trial, participants will first consent and download the POSTHOC (POST-Treatment Health Outcomes of Cancer Survivors) and Fitbit apps. After baseline assessments, they will be randomized and be in the study phase for 12 weeks. Our team will contact all participants at least every 2 weeks, and assessments will occur at weeks 6 and 12. We will then conduct an exit interview to solicit qualitative feedback on the app and the study.

**Figure 2:**
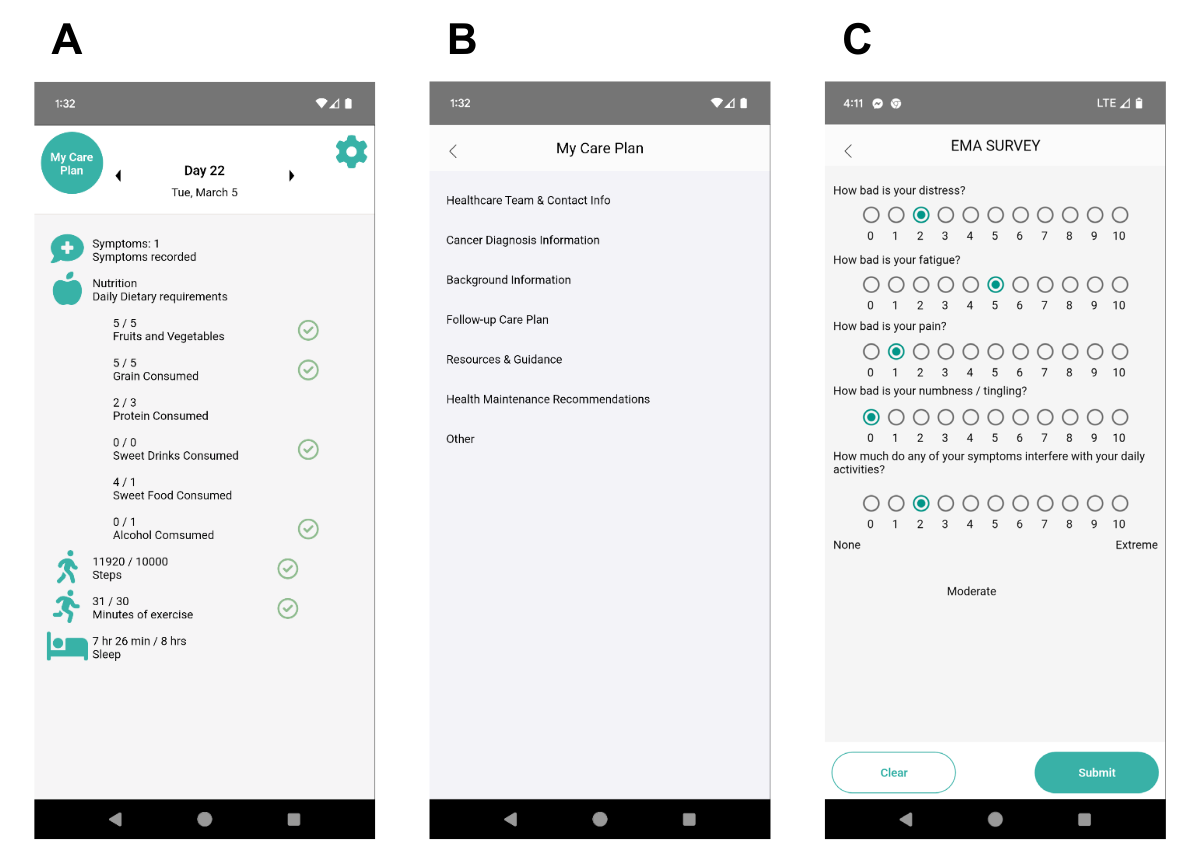
Screenshots of the POSTHOC (POST-Treatment Health Outcomes of Cancer Survivors) app. (A) The home screen for participants randomized to the POSTHOC arm of the clinical trial. Here, participants see whether they have recorded any symptoms and any dietary data they have entered. Steps, minutes of moderate and vigorous exercise, and sleep data are pulled from the Fitbit app. A green check indicates whether the goal was met for the day. Participants may navigate to the survivorship care plan (SCP; “My Care Plan”), Settings (gear in the top right), Symptoms, and Nutrition from here. (B) The SCP is divided into 6 sections based on the American Society for Clinical Oncology (ASCO) template for SCPs, survivorship guidelines from the National Comprehensive Cancer Network, and the University of Maryland Medical System’s SCP template. (C) Ecological momentary assessment (EMA) is administered 4 times per day for 7 days at baseline, week 6, and week 12. While the SCP will be manually entered into the POSTHOC app for this trial, it is an ultimate goal to interface the platform with the medical record so that the plan can be updated as a person’s health and goals change.

All study activities can be done remotely, and there is no essential face-to-face interaction between the study team and the participant (Figure 1). After consent, participants receive a Fitbit activity tracker (Charge 6) and orientation to use the tracker and POSTHOC app. The app and clinician portal are available through Charles River Analytics’ SparkCare platform. The app is freely downloadable from the Apple Store (Apple Inc) or Google Play (Google LLC). Participants need a personalized invitation from someone on our research team to set up an account in SparkCare and access the POSTHOC app. Our study team will guide the participant through the download and setup of Gmail (if they do not already have an account, which is necessary for Fitbit), Fitbit, and POSTHOC (Figure 2A [30-32]), either in person, over the phone, or via videoconference.

In addition, during baseline, participants will complete an On-Study form that includes questions on demographics and clinical characteristics and baseline questionnaires (approximately 35-50 minutes) via REDCap (Research Electronic Data Capture; version 13.7.4; Vanderbilt University). Participants will wear the activity tracker for 7 days to assess physical activity and sleep. We will also conduct EMAs via the POSTHOC app for these 7 days, during which participants will be prompted to answer 5 questions 4 times per day; the default times will be 9 AM, 12:30 PM, 4 PM, and 7:30 PM but can be adjusted based on the participant’s schedule and preferences (ideally begin 1 to 2 hours after waking; Figure 2C). These questions will capture key symptoms, such as distress, fatigue, pain, numbness and tingling, and interference of any symptoms with daily activity, on scales ranging from 0 (symptom not present) to 10 (as bad as you can imagine). Dietary intake data will be collected by a study team member using the Automated Self-Administered 24-hour (ASA24) Dietary Assessment Tool (National Cancer Institute) [33]. During the baseline week, all participants will have a “light” version of the POSTHOC app that has only initial data collection features, activity tracking and EMA data collection.

#### Randomization

We will use a computer-generated table with random blocks of 3 and 6 and stratify based on whether the participant had chemotherapy or not (yes or no) because symptoms and side effects of chemotherapy versus radiation tend to differ. The randomization table was generated by the statistician, SZ, and is stored in the REDCap database; allocation is concealed from the study staff who will be recruiting and randomizing the participants. After baseline data collection, participants will be randomized 2:1 to the POSTHOC app group (~36/54, 67%) versus the control group (18/54, 33%) in REDCap.

#### Delivery of the Intervention

If a participant is randomized to the intervention arm, a member of the study staff will switch them to the “intervention arm” in the clinician portal, and they will have full access to all the features of the app (Table 1). A trained member of the study team will enter data from the medical record into the SCP in the app (Figure 2B) and go over the SCP with them. The interventionalists will be trained and will use a checklist to ensure fidelity and prevent drift [34]. The participant will have a one-on-one meeting with an interventionalist and choose whether they want to prioritize nutrition or exercise as part of the study.

**Table 1:** Comparison of tools available to the intervention and control groups through the POSTHOC^a^ app.

Feature	Intervention	Control
Receive the SCP^b^	✓ (in the app)	✓ (on paper)
Ability to enter key symptoms during EMA^c^	✓	✓
Access to Fitbit	✓	✓
Access to the diet-tracking feature	✓	
Access to the comparison between their steps, minutes of moderate and vigorous exercise, dietary intake, and sleep time against the respective SCP guidelines	✓	
Location to log daily symptoms in the app	✓	

^a^POSTHOC: POST-Treatment Health Outcomes of Cancer Survivors.

^b^SCP: survivorship care plan.

^c^EMA: ecological momentary assessment.

Participants who choose to focus on exercise will be encouraged but not required to use their activity tracker during weeks 1 to 5 and 7 to 11 so that they can monitor their daily activity against their goals more easily. Over the course of the 12-week intervention, patients will gradually build up to goals laid out in the American College of Sports Medicine Physical Activity Guidelines for Cancer Survivors [35]. They will start at their baseline step level and increase their daily step goal by 5% to 10% each week (toward 10,000 steps per day, if possible). They will aim to complete 20 to 30 minutes of moderate-intensity aerobic exercise (40%-59% heart rate reserve) per day for a total of at least 150 minutes a week. They will also be encouraged to undergo stretching and resistance training for all major muscle groups 2 times a week, when possible.

Participants who choose to focus on nutrition will be encouraged to answer brief questions at the end of each day regarding their intake of fruits, vegetables, whole grains, and sweets to compare their eating habits to their goals in the POSTHOC app on a daily basis (Table S1 in Multimedia Appendix 2). Entry is possible for previous days if they forget to log. Goals will be based on the SCP guidelines, including working toward or maintaining a healthy BMI and consuming at least 5 servings of fruits and vegetables per day. Their goal will be to increase their adherence score for their nutrition goals, which will be determined through their questionnaire responses and the dietary assessment tool.

In the control arm, participants will keep the “light” version of the POSTHOC app that has minimal data collection features (Table 1). They will be told that they do not need to wear the activity tracker except for weeks 6 and 12. Participants will be provided with a digital (PDF) or paper-based version of the SCP, which they will go over with a trained member of the study team after randomization.

To match expectation effects across the 2 study arms, all participants will be told that we are testing a new app and that we are studying symptoms in early posttreatment survivorship. However, we will avoid the use of terms such as “control group” and “intervention group.” We will emphasize the importance of all participants’ data because we do not know much about the relationships between healthy lifestyle behaviors (eg, nutrition, physical activity, and sleep) and symptoms in early posttreatment survivorship.

For both arms, a member of the study team will call each participant approximately once every 2 weeks for the 12 weeks to encourage engagement; monitor adverse events; and, as appropriate, get feedback on the usability of the app (Table 2). At 6 and 12 weeks, the baseline assessments will be repeated, including the 35 to 50 minutes of questionnaires, wearing the activity tracker for 7 days, EMA for 7 days, and 24-hour dietary assessment. In total, we expect that participants will spend approximately 6 to 9 hours completing study activities over 14 weeks.

**Table 2:** Data collection table.

Assessment or activity			Study goal		Assessment location		Baseline (week 0)		Intervention (weeks 1-12)		Week 6		Week 12		Postintervention (week 13)	
**Coordinators only**																
	Clinical Record Form	Demographics and clinical characteristics		REDCap^a^		✓										
	Clinical Record Form	Health care utilization		REDCap						✓		✓				
	Clinical Record Form	Clinician-reported patient health (ie, ECOG^b^ performance status)		REDCap		✓						✓				
**Participants**																
	On Study^c^	Demographics and clinical characteristics		Clinic or home (REDCap^d^)		✓										
	Acceptability^c^ and usability^c^ of the POSTHOC^e^ app (intervention arm only)	Aim 1		Clinic or home (REDCap^d^)						✓		✓				
	MDASI^c,f^	Aim 2		Clinic or home (REDCap^d^)		✓				✓		✓				
	Activity tracker (Fitbit)	Aim 3		Home		✓				✓		✓				
	24-hour dietary assessment	Aim 3		Home		✓				✓		✓				
	GLTEQ^c,g^	Aim 3		Clinic or home (REDCap^d^)		✓				✓		✓				
	EMA^h^	Exploratory		Home		✓				✓		✓				
	FACIT-F^c,i^	Exploratory		Clinic or home (REDCap^d^)		✓				✓		✓				
	ISI^c,j^	Exploratory		Clinic or home (REDCap^d^)		✓				✓		✓				
	CIPN-20^c,k^	Exploratory		Clinic or home (REDCap^d^)		✓				✓		✓				
	BPI^c,l^	Exploratory		Clinic or home (REDCap^d^)		✓				✓		✓				
	BFI^c,m^	Exploratory		Clinic or home (REDCap^d^)		✓				✓		✓				
	DT^c,n^	Exploratory		Clinic or home (REDCap^d^)		✓				✓		✓				
	HCCQ^c,o^	Exploratory		Clinic or home (REDCap^d^)								✓				
	Health care Utilization	Exploratory		Clinic or home via phone						✓		✓				
	Telephone check-in (intervention and control)	Compliance booster and troubleshooting		Home				Biweekly for 12 weeks								
	Exit interview	Exploratory		Clinic or home										✓^p^		

^a^REDCap: Research Electronic Data Capture.

^b^ECOG: Eastern Cooperative Oncology Group.

^c^Questionnaire, as administered via a packet of questionnaires.

^d^If a participant prefers, they may complete paper-based versions of these assessments and return them to the study team in person or in a postage-paid envelope.

^e^POSTHOC: POST-Treatment Healthcare Outcomes for Cancer Survivors.

^f^MDASI: Monroe Dunaway Anderson Symptom Inventory.

^g^GLTEQ: Godin Leisure-Time Questionnaire.

^h^EMA: ecological momentary assessment.

^i^FACIT-F: Functional Assessment of Chronic Illness Therapy–Fatigue.

^j^ISI: Insomnia Severity Index.

^k^CIPN-20: Chemotherapy-Induced Peripheral Neuropathy–20.

^l^BPI: Brief Pain Inventory-Short Form.

^m^BFI: Brief Fatigue Inventory.

^n^DT: Distress Thermometer.

^o^HCCQ: Health Care Climate Questionnaire.

^p^The exit interview will be conducted earlier than week 13 if the participant withdraws early.

### The POSTHOC App

The POSTHOC app was designed by Charles River Analytics specifically for this project (screenshots in Figure 2). It was designed to run on both Android (Google LLC) and iOS (Apple, Inc) devices, which account for >90% of all smartphones in the United States [36]. A dedicated team of developers will make periodic updates to the app and to the backend server to comply with updates released by Android and Apple. The trial opened in March 2024 with version 1.1.11, and as of July 2024, the recent version was 1.1.13. Updates were made to increase the speed at which the app syncs with Fitbit and opens for the user.

The app was designed to (1) store information from the SCP, including information about diagnosis, treatments, and future plans and (2) collect data regarding ongoing health status, as it pertains to the SCP (eg, nutrition, physical activity, sleep habits, and symptoms). Data will be collected through a combination of manual entry and automatic collection by the Fitbit activity tracker that we will provide. All data collected by the POSTHOC app (ie, physical activity, diet, and EMA data) will be stored on a Health Insurance Portability and Accountability Act (HIPAA)–compliant server at UMB.

### Outcome Measures

#### Physical Activity, Diet, and Symptoms

Physical activity and sleep will be measured via a Fitbit Charge 6, which will be given at no cost to the participants. They will be asked to wear the Fitbit for 7 days during weeks 0 (baseline), 6, and 12. Dietary intake will be assessed using the ASA24 Dietary Assessment Tool [33]. The ASA24 Dietary Assessment Tool is a freely available web-based tool used for epidemiologic, interventional, behavioral, or clinical research that enables multiple automatically coded 24-hour dietary recalls. It will be administered by a licensed dietitian or registered nurse in person, via telephone, or via videoconference and takes approximately 30 to 45 minutes.

EMA will be collected via the POSTHOC app 4 times throughout the day for 7 days at weeks 0, 6, and 12. The app will alert the participant to report the severity of 4 common symptoms, namely pain, distress, fatigue, numbness or tingling, and the interference of symptoms with daily activity on a scale of 0 to 10. While research studies have shown success with this type of data collection [37], participants will have the option to “turn off notifications” if they are too burdensome. The POSTHOC app does not have any notifications other than those for the EMAs.

#### Questionnaires

Questionnaires will be administered via REDCap [38] or on paper, and then the responses will be manually entered into REDCap at weeks 0, 6, and 12 (Textbox 1). There are 15 questionnaires; 13 are self-administered, and 2 are researcher-administered. The On Study form will be completed at baseline only, questionnaires on Health Care Utilization and the Acceptability and Usability of the app will be administered during a routine check-in call at weeks 6 and 12 only, the Health Care Climate Questionnaire will be administered at 12 weeks only, the questionnaire on Acceptability and Usability will be administered to the intervention group only, and the 11 other questionnaires will be completed at baseline, week 6, and week 12 (Table 2). They should take approximately 35 to 40 minutes in total to complete.

Responses to questionnaires (eg, On Study), as well as the Clinical Record Form, will be collected via REDCap and, therefore, will be stored on UMB servers. Charles River Analytics will not have access to any of the data collected in this study.

**Questionnaires to be administered.**

**Questionnaires**

1. On Study: this form contains questions on demographics; clinical characteristics; and how much participants think the app, exercise, and healthy eating could help reduce any symptoms they are experiencing. This is a custom questionnaire that should take 5 to 10 minutes to complete.
2. MD Anderson Symptom Inventory (MDASI) [39-41]: the 13-item MDASI is a patient-reported multisymptom outcome assessment scale that assesses the severity of symptoms experienced by patients with cancer and how much the symptoms interfere with daily living (eg, walking, activity, working, housework, relations with other people, enjoyment of life, and mood). The assessment takes ≤5 minutes to complete. The Cronbach α reliability ranges from 0.82 to 0.94. The sum of the 13 items will be used as the primary aim herein.
3. Functional Assessment of Chronic Illness Therapy–Fatigue (FACIT-F) [42]: the FACIT-F is a 40-item, validated, self-administered, and commonly used measure of fatigue and quality of life over the last 7 days with 5 subscales, namely physical well-being, social well-being, emotional well-being, functional well-being, and fatigue. A higher score indicates a higher quality of life. The assessment takes 10 to 15 minutes to complete. The Cronbach α reliability is 0.96.
4. Brief Fatigue Inventory (BFI [43]): the BFI is a 10-item, validated, self-administered, and commonly used measure that captures 24-hour fatigue and includes 6 single-item questions regarding how fatigue has interfered with general activity, mood, work, etc. The assessment takes ≤5 minutes to complete. The Cronbach α reliability ranges from 0.82 to 0.97.
5. Brief Pain Inventory–Short Form (BPI-SF) [44]: the BPI is a self-administered, validated measure of pain severity at its least, worst, and average in the last 24 hours from 1 to 10. It enquires about pain right now; medications taken for pain; and how the pain has interfered with general activity, mood, walking, work, relations with other people, sleep, and the enjoyment of life. The assessment takes ≤5 minutes to complete. The Cronbach α reliability ranges from 0.77 to 0.91.
6. Insomnia Severity Index (ISI) [45,46]: the ISI is a validated self-administered 7-item questionnaire that assesses sleep quality based on difficulties falling asleep, staying asleep, and waking up too early; satisfaction with sleep pattern; how noticeable any sleep problem is to others; worry about sleep problems; and interference of sleep problems with daily functioning. The assessment takes ≤5 minutes to complete. The Cronbach α reliability is 0.79.
7. Godin Leisure-Time Exercise Questionnaire (GLTEQ) [47]: the GLTEQ is a 3-item self-administered questionnaire that measures the frequency of strenuous (eg, running, hockey, vigorous swimming, roller-skating, and judo), moderate (eg, fast walking, easy swimming, easy bicycling, volleyball, and badminton), and mild or light (eg, yoga, fishing from the river bank, easy walking, and bowling) leisure-time physical activity performed for periods of ≥15 minutes over a usual week, with resulting scores interpreted as active, moderately active, or insufficiently active or sedentary.
8. Chemotherapy-Induced Peripheral Neuropathy-20 (CIPN-20) [48]: the CIPN-20 is a 20-item self-administered quality-of-life questionnaire to elicit patients’ experience of symptoms and functional limitations related to numbness, tingling, and other symptoms of chemotherapy-induced peripheral neuropathy and is recommended by a National Cancer Institute (NCI) task force [49]. The Cronbach α reliability ranges from 0.73 to 0.88.
9. Functional Assessment of Cancer Therapy-Cognition (FACT-Cog) [50]: the FACT-Cog is a 37-item self-assessment scale validated in routine clinical practice to assess chemotherapy-induced cognitive impairment in adults with cancer. The assessment takes 10 to 15 minutes to complete. The Cronbach α reliability ranges from 0.58 to 0.63.
10. Hospital Anxiety and Depression Scale (HADS) [51]: the HADS is a 14-item self-reported questionnaire that comprises 7 questions for depression and 7 questions for anxiety and takes 2 to 5 minutes to complete. The Cronbach α reliability is 0.78 for the anxiety subscale and 0.86 for the depression subscale.
11. Distress Thermometer (DT) [52]: the DT is a common self-reported rapid screening tool that uses a rating scale ranging from 0 (no distress) to 10 (extreme distress) and was developed to assess psychological distress in people affected by cancer. The assessment takes approximately 2 to 3 minutes to complete.
12. Health Care Climate Questionnaire (HCCQ) [53] (modified; 12 weeks only): we modified the HCCQ to probe communication regarding healthy lifestyle behaviors (eg, Your doctor encouraged you to ask questions about healthy lifestyle behaviors). This questionnaire includes 6 questions, each with response options ranging from 1 (strongly disagree) to 5 (strongly agree); the total score ranges from 6 to 30, with 6 indicating the lowest satisfaction with patient-provider communication and 30 indicating the highest satisfaction.
13. Health Care Utilization survey (6 and 12 weeks only): here, we will inquire about any medical appointments (scheduled or unscheduled), hospitalizations, out-of-pocket health care expenses, and whether any of the events were cancer related.
14. Acceptability survey (6 weeks and postintervention interview only): we will ask 6 questions to gather feedback about how much the participant liked the app, what features they enjoy or do not use, and whether they would recommend the app to others.
15. System Usability Scale (SUS) [54,55] (6 weeks and postintervention interview only): the SUS is a simple questionnaire designed to assess the usability of a particular device or product on a Likert scale of 1 (strongly agree with the statement) to 5 (strongly disagree with the statement).

#### Exit Interview

After the completion of the study (or earlier if a participant withdraws early from the study), a member of the study team will conduct an exit interview to understand the participants’ experience of being in the study. We will ask them whether they think the app was helpful for alleviating their symptoms and improving their health behaviors. We will ask them what they liked about the study, what they did not like about it, what features they liked or did not like about the POSTHOC app (for those in the intervention arm), whether they would recommend the study to others, etc. To get more information regarding patient-provider communication, we will ask about the number and quality of conversations regarding physical activity and diet between a patient and their provider or providers, as well as questions to understand patients’ preferences and expectations in regard to where (eg, clinic or home), when, how often, and with whom (eg, primary care physician or oncologist) patient-provider communication regarding lifestyle behaviors should take place. This semistructured interview will use qualitative methods and will last approximately 30 minutes [56]. It will be audio recorded with explicit permission at the time of the interview and then transcribed for qualitative and mixed methods data analyses.

For qualitative analysis, we will use the qualitative descriptive approach [57]. This approach solicits information from those experiencing the phenomenon under investigation, that is, the use of the POSTHOC app in early posttreatment cancer survivorship. We will analyze data from both the POSTHOC group and the control group, although the control group will not be asked some questions that are specific to the app. Our analysis will involve a deductive approach, where codes will be based on the questions in the exit interview. In addition, we will use a secondary inductive approach to capture themes in the data on which questions were not originally included in the interview guide [58,59]. With an iterative process, at least 2 independent coders will review all the transcripts and use deductive coding in response to questions asked about specific elements of the POSTHOC app, the study, etc, as well as open coding to inductively identify emergent themes. All reviewers will then convene with the study chair to discuss codes and subcodes, standardize them, and then generate a codebook. Coders will examine the transcripts a second time, and then, the team will reconvene and refine the codes as appropriate. Coding will then be completed. The total number of times a code was used, as well as the relative proportion of participants who had a quote that fit into each code, will be quantified and compared, and differences will be reconciled through discussion.

#### Adherence

We will measure adherence to the intervention, retention, requests for data entry, and adherence to the goals of the intervention in relation to what is laid out in the SCP. Retention will be measured based on how many participants complete week-12 questionnaires; we predict that we will have ≥85% retention. Adherence to the study requests will be monitored as the percentage of data points received version requested, which will be different for those in the POSTHOC arm versus the usual care arm and for those in the nutrition group versus exercise group within the POSTHOC arm. We predict that we will have lower response rates in the groups that are asked to contribute more (ie, in terms of daily nutrition entries). Nutrition adherence will be measured with an adherence score calculated at week 6 and week 12 based on participant responses to brief questions asked at the end of each day regarding their intake of fruits, vegetables, whole grains, and sweets. Their score will be compared to goals in the SCP. For those who choose to emphasize exercise, actual physical activity from Fitbit will be compared to physical activity goals outlined by the SCP.

#### Medical Records

The Clinical Record Form will be completed by the study staff at baseline. It contains questions on demographics, height, weight, current menopausal status, Karnofsky performance status or Eastern Cooperative Oncology Group performance status, comorbidities (eg, hypertension and diabetes), cancer type, cancer stage, surgical procedures, types and doses of treatments (eg, chemotherapy type and dosing, hormone therapy, and radiotherapy regimen), most recent blood work (eg, hemoglobin, hematocrit, and lymphocytes), other medications, and medical history (eg, prior myocardial infarction).

At weeks 6 and 12, a member of the study team will record Health Care Utilization, including the number of unplanned medical encounters such as emergency department, urgent care, oncologist, and primary care visits over the previous 6 weeks (approximately 1 and a half months). At week 12, we will note the provider-rated Eastern Cooperative Oncology Group status (or equivalent).

### Statistical Analysis

#### Data Handling

We will capture patient-reported data electronically using the POSTHOC app and REDCap [38]. Data from the POSTHOC app will be viewable on the clinician portal and exportable to a CSV file; REDCap data will also be exportable to a CSV file. Study data will be deidentified for analysis. We will audit our database and visually inspect all data (eg, Q-Q plots, boxplots, and scatter plots). These inspections will include histograms of various outcome variables as well as plots of means and fitted regression curves. If distributional assumptions are not met (eg, normality of residuals), we will use transformations or nonparametric methods [60]. We will assess outliers to determine whether they are erroneous. If they are valid, we will conduct sensitivity analyses with and without outliers.

We will facilitate the completion of all measures. The reasons for missing data will be tabulated according to the treatment group (via interview, if possible), and participants who dropped out will be compared with those who completed the study with regard to demographic and disease characteristics. The mechanism of missing data will be assessed to select an appropriate statistical method [61-63]. Specifically, if the data appear to be missing at random, we will use multiple imputation or the maximum likelihood method to obtain unbiased estimates of key statistics. If the data are suspected to be missing not at random, a sensitivity analysis using selection and pattern mixture models will be run to determine the impact on results. If the estimates are similar to the ones obtained from the simpler analysis of only complete cases, we will report the complete-case analysis results.

#### Sample Size

Power calculations were based on data from a prior single-arm pilot study that we conducted among a similar group of cancer survivors (National Institutes of Health project 75N91019C00026). Results from this analysis of covariance suggested a pre-post correlation of 0.65 [64]. With 2-tailed α=.15 [49], a sample size of 46 (after 15% missing data and a 2:1 ratio of sample in arms) would provide 80% power to detect a standardized between-arm difference of 0.50, which corresponds to a medium-sized and clinically meaningful effect [65]. Preliminary data generated from another previous study of ours among cancer survivors [66] suggested an SD of cumulative symptom burden from a similar symptom inventory of 17.0. On the basis of the estimated effect size for this study, 0.50, we expect that the average cumulative symptom burden will decrease by 8.5 points more in the POSTHOC group than in the control group from baseline to 12 weeks.

#### Primary Outcome

The primary outcome is the feasibility of the POSTHOC app, which will be determined by calculating the percentage of patients with cancer in the POSTHOC group that used the app at least 3 times per week. We hypothesize that at least 75% (≥27/36) of patients will view or log data in the app at least 3 times per week at weeks 6 and 12.

#### Statistical Analysis for Aims

The statistician cannot be blinded due to the 2:1 randomization scheme.

Aim 1 assesses feasibility, acceptability, and usability. For feasibility, we will calculate the percentage of patients who used the POSTHOC app at least 3 times per week, as explained earlier. For acceptability, at weeks 6 and 12, we will estimate the percentage of patients who (1) found the app useful (≥4 on a scale of 1 to 7), (2) would recommend it to others (≥4 on a scale of 1 to 7), and (3) would use the app in the future (at least monthly). For usability, we will calculate summary statistics based on an overall usability score from the System Usability Scale (score range 0-100) [54]. A score of 68 (12.5) is considered average, with >68 points considered “above average” and <68 “below average” [55]; these values are relevant to digital health apps [67]. We will review the qualitative data within the acceptability or usability questionnaires and those collected during interviews to identify general themes related to specific design features that participants liked or that make the app challenging to use or understand.

Aim 2 assesses the effects of the intervention versus control on cumulative symptom burden. Our data from aim 2 will provide estimated effect sizes to inform the sample size of a confirmatory phase III randomized controlled trial, per clinical trial guidelines [68]. Cumulative symptom burden will be calculated as the sum of the individual severity levels from the 13 core symptoms from the MD Anderson Symptom Inventory (total symptom burden ranging from 0 to 130) [39,40]. We will use linear mixed modeling [69] with total symptom burden at week 6 as the primary symptom outcome, study arm (POSTHOC app vs usual care arm) as the main independent variable, and total symptom burden at week 0 as a covariate. In the case of a substantial imbalance in other factors between arms at baseline (eg, age), we might include additional covariates in the model. We chose week 6 as the primary time point because preliminary data from our group show greater benefits of behavioral interventions earlier into the interventions rather than later (IR Kleckner, unpublished data, August 2024; and Kleckner et al [70]). We will use the intent-to-treat principle and include each participant in the arm to which they were assigned, regardless of the use of the app for those assigned to the POSTHOC arm. We will also assess the effects of the intervention versus usual care at week 12 as an exploratory aim.

Aim 3 will evaluate the effects of the intervention on physical activity (steps and time engaged in moderate and vigorous physical activity) and nutrition (ASA24 scores and daily habits). Similar linear mixed models will evaluate the effects of the POSTHOC app versus usual care on other outcomes at week 6 and week 12. To evaluate the longitudinal trajectory of each outcome, we will use repeated measures linear mixed models incorporating the specific outcome at all 3 time points and using a linear contrast to test the effects of study arm.

Exploratory outcome analyses will include a test of mediation and moderation effects of the POSTHOC app by physical activity adherence, sex, age, etc on outcomes [71-73].

## Results

This project was funded by the National Cancer Institute in September 2021. Approval from the Institutional Review Board at the UMB was obtained on February 21, 2024, and the trial was opened to accrual on March 1, 2024. As of July 3, 2024, a total of 20 participants have been enrolled. We expect to complete enrollment in fall 2024, complete data collection in winter 2025, and publish the results in winter 2026.

## Discussion

### Expected and Predicted Results

In regard to our first hypothesis that the POSTHOC app is feasible, acceptable, and useful for cancer survivors, we predict that at least 75% (≥27/36) of participants in the POSTHOC arm will view or log data in the app at least 3 times per week. We also predict that at least 75% (≥27/36) of patients will enjoy using the app (Likert rating of >3 out of 5) and will recommend it to other patients (yes or no) at weeks 6 and 12. We further expect that participants will find the app useful overall but will suggest areas for improvement. In regard to our second hypothesis that POSTHOC will reduce the cumulative symptom burden reported by patients compared to usual care, we predict that the sum of the 13-item MD Anderson Symptom Inventory will be at least 8.5 points lower for patients in the POSTHOC arm than for patients in the usual care arm. Finally, in regard to our third hypothesis that the POSTHOC app will improve health behaviors compared to usual care, we predict that patients in the POSTHOC arm who choose to focus on exercise will complete at least 500 more steps per day than patients in the usual care arm (but hopefully at least 1000 to 2000 steps more per day than controls), based on our prior studies of exercise in patients with cancer [74]. We predict that these extra daily steps will correspond to an extra 10 to 20 minutes per day of moderate physical activity; thus, more patients in the POSTHOC group will meet the American College of Sports Medicine Physical Activity Guidelines for Cancer Survivors [35] than patients in the control group. For participants who choose to focus on nutrition, we predict that they will have a higher adherence to the nutrition goals laid out in their SCP than participants in the control group based on data from the 24-hour dietary assessment. We will also explore whether those in the POSTHOC arm who focused on exercise adhered to the nutrition goals laid out in the SCP compared to those in the control arm and compared to those who chose to focus on nutrition.

### Integration of This Project Into Prior Literature

The growing population of cancer survivors must cope with symptoms of the disease and side effects of treatment, and current SCPs are not enough to ensure timely symptom identification and adherence to recommendations in the SCPs [8]. They are burdensome, complex, and not easily accessible. Despite increasing interest in mHealth interventions to enhance overall wellness (eg, Fitbit and MyFitnessPal [MyFitnessPal, Inc]) and the development of apps to store the SCP (eg, the studies by Baseman et al [20], Preussler et al [21], and Denzen et al [22]), no platform has capitalized on the potential of mHealth to combine the SCP with healthy behavior monitoring. This protocol describes a randomized controlled trial to evaluate the feasibility, usability, and preliminary efficacy of a new mobile SCP app that interfaces with Fitbit, POSTHOC, versus a traditional paper-based SCP in terms of total symptom burden in the early posttreatment period in addition to its ability to promote healthy lifestyle behaviors and patient-provider communication.

Charles River Analytics completed a phase I project to launch the development of an app called POSTHOC to digitize the SCPs. Specifically, the team conducted targeted interviews with survivors, clinicians, and researchers to understand how providers track symptoms of cancer with their current tools, including the SCP, and what features could be added to the existing tools and workflows to streamline care. Accordingly, the POSTHOC app was designed to go beyond routine plans to deliver an updatable SCP, collect data regarding ongoing health status, and promote healthy lifestyle behaviors. The success of the phase I of the project enabled us to build upon the platform in phase II, which involves the testing of the app in a clinical population regarding feasibility, usability, and preliminary efficacy in reducing symptom burden.

Prior studies with digital SCPs have not used a care plan–specific mobile app, have been underpowered to detect changes in physical activity patterns, or have tended to focus exclusively on exercise interventions rather than nutrition and exercise interventions [20-22,75,76]. If successful, this study will contribute to the user-centered design of a platform for a digital SCP. In addition, it can assist cancer survivors with engaging in healthy behaviors and facilitate communication between the survivor and the person’s clinical care team, including their primary care physician. This study will provide evidence about the effects of the self-management of health on health care use, cancer symptoms, and treatment side effects. This knowledge will be integral to later, larger randomized controlled studies; integration with the electronic medical record; and nationwide or worldwide implementation.

### Clinical Implications

In the near future, the POSTHOC app could help advance the management of post–cancer treatment symptoms with nutrition and exercise interventions. We foresee 3 key benefits to the integration of the POSTHOC app into the clinical workflow. First, the app will be able to gather personalized health information outside the clinic’s walls and provide on-demand, real-time feedback to survivors. A future goal is for the app to be able to interface with medical records to facilitate conversation about health-promoting behaviors between the patient and the clinical care team. These personalized health data permit providers to keep track of emerging symptom patterns and adherence to the SCP and provide support tools as they seek to promote healthy behaviors. Second, the app will provide information and tools to health providers in an easy-to-use format, giving health care professionals unprecedented access to survivor data to guide patient-provider communication and clinical decision-making, including oncologists, primary care physicians, and physical therapists, as we have suggested for treating chemotherapy-induced neuropathy [77]. Finally, the app will securely handle personalized health information to promote accessibility while maintaining interoperability with hospital information technology. This increases provider efficiency while maintaining the focus on survivor needs.

It is the ultimate goal for the POSTHOC app to be available to anyone with an Apple or Android smartphone. While the features of the app would remain the same between real-world and research settings, there would be one main difference in the setup: the research team is currently manually entering the SCP into the app for the participant. In the real world, the clinical care team should not be burdened with entering the SCP into a separate app, but the goal would be for the app to interface with the electronic medical record so that the app can automatically fetch the SCP and continuously update as the user’s health status and goals change.

### Potential Problems and Solutions

We are prepared to address potential problems that may arise during the implementation of the study. During the recruitment phase, we may have trouble recruiting participants. We will then widen our eligibility criteria and use different advertising strategies, including forming closer relationships with oncologists and advanced practice providers, spending more time with patients in clinics, and advertising among cancer support groups. In the intervention phase, participants may have difficulty understanding the user interface and using the app. We will do our best to provide comprehensive guidelines for use. If necessary, we will offer more opportunities for in-person instructions and weekly tutorials with study team members. In addition, participants may not use the app and answer survey questions regularly. To encourage participants to actively engage with the app whenever possible, we will plan consistent check-ins with study team members. If technical issues arise, a support team from Charles River Analytics get on call to push updates wirelessly and address technological concerns.

### Strengths and Limitations

This study has several strengths. First, we expect this study to include a diverse sample of participants and have high generalizability to cancer survivors in the United States. In the UMGCCC catchment area, approximately 30% of people identify as Black or African American, compared to 13% of Americans across the United States, and we expect our cohort to reflect our catchment area. Moreover, we will recruit from the cancer center in downtown Baltimore, Maryland, as well as from affiliated community oncology clinics in suburban and rural areas. Second, this study uses EMA and ultrahigh-frequency sampling, with data collected daily during the morning, early afternoon, later afternoon, and evening. The results may offer insights into the interplay of exercise, symptom burden, and circadian effects. They can also be applied to make inferences regarding causality on a small-time scale (ie, throughout the day and day to day). Past studies have either been underpowered to detect daily changes in physical activity or used lower-frequency sampling, asking participants to respond to 1 or 2 daily messages or a set number of prompts within a designated period [75,76]. Third, this study offers both nutrition and physical activity options. Prior studies have tended to focus exclusively on exercise rather than nutrition and exercise interventions and have not offered participants the option to focus on either exercise or nutrition.

This study also has limitations. First, this is a phase II feasibility and preliminary efficacy study (n=54), and the results should be tested for replication and validation before being implemented into guidelines. Nevertheless, we expect that this work will help lay the groundwork for larger studies to test similar hypotheses. Second, this study includes a usual care control instead of an active control. However, in this context, including a usual care control allows for the most direct evaluation of a mHealth-delivered behavioral intervention in comparison to an mHealth diet and activity tracker. Third, this study collects information primarily from patient-reported outcomes, with no clinical assessments of signs (eg, blood-based biomarkers). However, the data collection methods include digital measures of diet, activity, and sleep, which allow for objective assessments of physical health. Fourth, bias may be introduced into this study in that only people who are comfortable enough with technology to beta-test an app will enroll. Those who enroll and are less comfortable with technology might be more likely to remain in the study if they are in the control group than if they are in the POSTHOC group, but dropout rates should not affect our primary outcome (feasibility, acceptability, and usability, analyzing only data from the POSTHOC arm) or symptom data in aim 2. While we might have POSTHOC participants who do not use the app (nonadherence), it will not be possible for control participants to use the app (contamination) because we have control over what features all the participants can see. Nonadherence will lead to more conservative effect size estimates of the effect of the intervention on symptom burden. Our analyses will be a step toward understanding how we can digitize the SCP into a mobile app to help improve outcomes for posttreatment cancer survivors.

### Future Work

Future initiatives will work to optimize the reach and usefulness of the POSTHOC app, as well as the design and logistics of clinical trials to test the app’s efficacy in alleviating symptom burden. For example, we are working toward allowing the POSTHOC app to interface with the electronic medical record to facilitate conversations about lifestyle behaviors between the patient and the clinical care team. We will also incorporate feedback from participants and clinicians to improve the features of the app to maximize its usability and usefulness for survivors, their caregivers, and their clinical care team.

## Appendix

Multimedia Appendix 1 Consent form.

Multimedia Appendix 2 Supplementary material on the consent process, privacy, confidentiality, and the daily nutrition questions.

Multimedia Appendix 3 CONSORT-eHEALTH submission form.

Multimedia Appendix 4 Peer-review from Small Business Innovation Research (SBIR) - National Institutes of Health (NIH) and the Centers for Disease Control and Prevention (CDC) Proposals (USA).

## Abbreviations

ASA24: Automated Self-Administered 24-hour
EMA: ecological momentary assessment
HIPAA: Health Insurance Portability and Accountability Act
INSPIRE: Internet Survivorship Program with Information and Resources
mHealth: mobile health
POSTHOC: POST-Treatment Health Outcomes of Cancer Survivors
REDCap: Research Electronic Data Capture
SCP: survivorship care plan
UM: University of Maryland
UMB: University of Maryland Baltimore
UMGCCC: University of Maryland Greenebaum Comprehensive Cancer Center

## Data Availability

The source codes of the POSTHOC (POST-Treatment Healthcare Outcomes for Cancer Survivors) app and SparkCare server are owned by Charles River Analytics and are confidential and proprietary under NIH SBIR 75N91021C00019. Researchers who would like to replicate, build upon, or collaborate on research involving these systems are encouraged to reach out to Charles River Analytics [78]. Deidentified data collected as part of the clinical trial may be shared with a data use agreement with the University of Maryland Baltimore upon reasonable request to the corresponding author, ASK.
