# Supplementary material for: Digitizing Survivorship Care Plans Through the POST-Treatment Health Outcomes of Cancer Survivors (POSTHOC) Mobile App: Protocol for a Phase II Randomized Controlled Trial
Source: JMIR Res Protoc. 2024 Sep 5;13:e59222. doi: 10.2196/59222 (PMC11413545; doi:10.2196/59222)
Supplement: Multimedia Appendix 1 [file resprot_v13i1e59222_app1.pdf]

UNIVERSITY of MARYLAND
BALTIMORE

RESEARCH CONSENT FORM AND HIPAA AUTHORIZATION

Protocol Title:
Leveraging technology to address health outcomes of cancer survivors (POSTHOC)

Study No.: HP-00100473

Principal Investigator: Amber Kleckner, PhD, 410-706-5961

Sponsor: National Cancer Institute (NCI), National Institutes of Health (NIH), United States Department of Health and Human Services (HHS)

This Consent document describes a research study, what you may expect if you decide to take part, and important information to help you make your decision. Please read this form carefully and ask questions before you agree to participate.

CONCISE SUMMARY:

At the end of cancer treatment, many people are still dealing with symptoms of cancer and side effects of treatment. Survivorship Care Plans are plans that are provided to individuals at the completion of cancer treatment and describe the details of a person's diagnosis and treatment, as well as provide recommendations for follow-up appointments, referrals, and healthy behaviors to quicken recovery and prevent the cancer from coming back. In this study, we will be testing the effects of the established paper-based Survivorship Care Plan vs. a new smartphone app on symptoms and mood in early post-treatment survivorship.

The study lasts approximately 14 weeks. At the beginning of the study, you will be asked to:

- Complete online questionnaires related to your symptoms and feelings,
- Have a phone call or video session (your choice) with a study team member to talk about your typical dietary habits and explain the study,
- Wear a Fitbit smartwatch on your wrist to assess your physical activity and sleep, and
- Answer five questions, four times per day, about key symptoms (you pick the times, for example 9am, 12:30 pm, 4 pm, and 7:30pm), via a smartphone app that we provide.

After the first week, you will be randomly assigned to one of two groups: a paper-based or app-based Survivorship Care Plan. Those in the app group will choose to focus on nutrition or exercise for the duration of the study. At the middle (week 6) and end (week 12) of the study, we will ask you to complete the same study activities as at the beginning (online questionnaires, diet, wear the Fitbit, answer the symptom questions). Study materials may be provided in-person or via mail – your choice (i.e., you do not have to come in). You will be paid a total of $50 for your time to complete the study activities, and you can keep the smartwatch.

Key risks: emotional distress; breach of confidentiality; injury from physical activity.

Participating in this research study is voluntary. Your decision to participate will not affect your healthcare or treatment for your cancer in any way.

CONTACT INFORMATION:

Study team: nrsPOSTHOC@umaryland.edu
Principal investigator: Amber Kleckner, PhD, (410) 706-5961, amber.kleckner@umaryland.edu

HP-00100473 UM IRB Approval Date 5/23/2023

PURPOSE OF STUDY

In this study, we want to test the effects of the traditional paper-based Survivorship Care Plan compared to a new smartphone application (“app”), “POST-Treatment Health Outcomes of Cancer survivors” or “POSTHOC,” on overall symptom burden in the early post-treatment cancer survivorship period. We want to collect feedback on the app’s features so that it can maximally help people transition between active cancer treatment and early survivorship. Also, we want to get more information on the relationships between lifestyle behaviors (i.e., nutrition, physical activity, and sleep) and common cancer-related symptoms such distress, fatigue, pain, numbness/tingling, and how much these symptoms interfere with daily life; this will allow us to design more effective survivorship programs.

Approximately 54 participants will be recruited to take part in this study from the University of Maryland Medical System.

PROCEDURES

Here are the study activities and timeline. Each of the activities is described in more detail below.

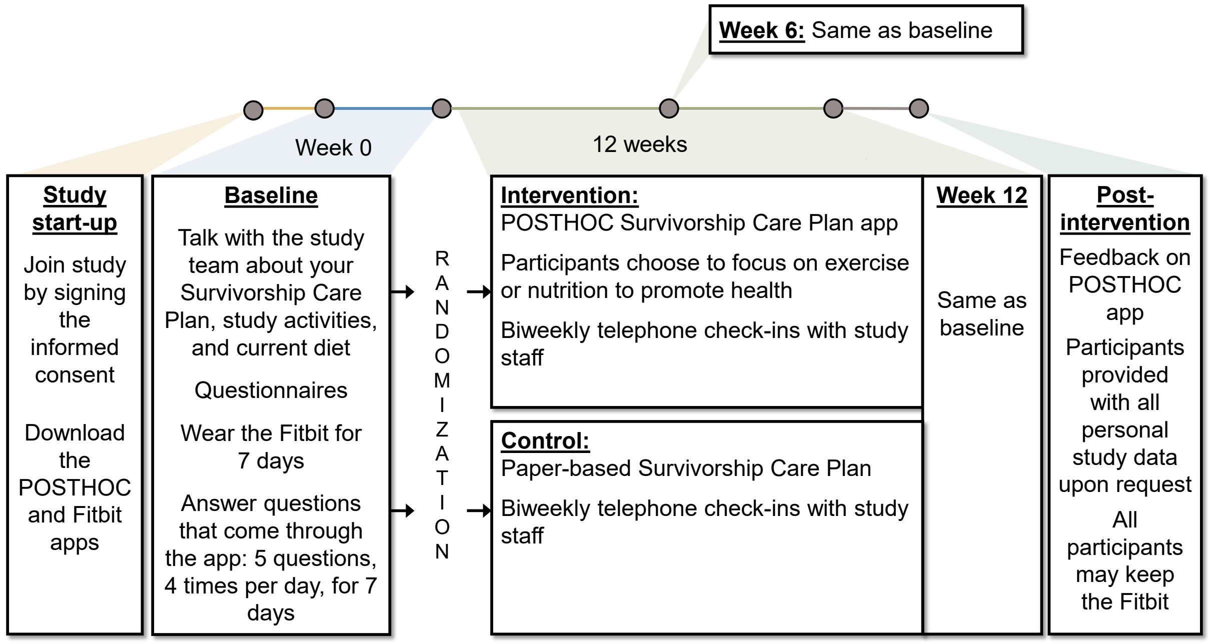

The Survivorship Care Plan: Everyone in the study will receive a Survivorship Care Plan, either on paper, which is standard, or via the app, if you are in the app group. Specifically, the Survivorship Care Plan will list the names and roles of your providers, including your oncologist, primary care provider, and any specialists you have. It will list upcoming appointments and recommendations for future appointments with the target time frames (e.g., annual mammogram for breast cancer, annual CT scan for colorectal cancer). The Survivorship Care Plan also includes healthy lifestyle behavior goals for nutrition, exercise, sleep hygiene, and quitting tobacco products.

HP-00100473 UM IRB Approval Date 5/23/2023

Fitbit: The Fitbit is a smartwatch that is used to track physical activity and sleep patterns. We will ask you to wear a Fitbit on your wrist every day for weeks 0, 6, and 12. We will help you set up the Fitbit app, and the POSTHOC app will automatically pull your activity data from the Fitbit app; no activity data will need to be entered by you. The Fitbit is not intended to be used as a medical device.

Questionnaires: We will ask you to complete 9-11 online questionnaires (depending on the time point) that ask about your symptoms, feelings, and habits. These will take approximately 35-50 minutes to complete—you can do them all at once or spread them out throughout the week. You also have the choice to complete these on paper. We will ask you to complete questionnaires three times throughout the study—at weeks 0, 6, and 12.

24-Hour Dietary Recall: A member of our study team will perform a 24-hour dietary recall with you during either a phone call or video session (your choice) three times throughout the study period. This will take approximately 30-45 minutes to complete.

Ecological Momentary Assessment: At four time points every day for seven days (at weeks 0, 6, and 12), we will "ping" you through the POSTHOC app to report the severity of four common symptoms—distress, fatigue, pain, numbness/tingling—and how much these symptoms are interfering with your daily activities on a scale of 1-10. We expect that you respond promptly (within 10 minutes or so) to the notifications.

Randomization and the two study groups: After the first week, you will be randomly assigned to one of two groups: the digital Survivorship Care Plan (i.e., the "POSTHOC app") group or the paper-based Survivorship Care Plan (i.e., the control group). No one on the study team knows which group you will be in until after week 0. The group you will be in is chosen by chance, like drawing a number out of a hat. There is a two-thirds (67%) chance you will be in the POSTHOC app group and a one-third (33%) chance you will be in the control group.

- POSTHOC app group: If you are assigned to the app group, the study team will input your Survivorship Care Plan into the app. You will choose whether you would like to prioritize nutrition or exercise as part of the study. We will set goals with you based on your Survivorship Care Plan, and teach you how to read and understand either the exercise data or food log data in the app. If you choose to focus on exercise, you will be encouraged to wear your Fitbit for the duration of the study (not just weeks 0, 6, and 12). If you choose to focus on nutrition, you will be encouraged to answer a few short questions regarding your diet at the end of each day, which will take approximately 30-60 seconds. In total, the time commitment will be a few minutes per week. You will also have access to other features of the app, such as logging symptoms.
- Control group: If you are assigned to the control group, you will not be asked to make any changes to your diet or exercise pattern.

Contact with the study team: We will call you about every two weeks to check in and see how you are doing. You may also call or email the study team in between these check-ins if you have any questions or concerns.

Exit interview: At the completion of the study, we will "interview" you about your experience in the study. We will ask you what you liked about it, what you didn't like about it, and ask for feedback on the POSTHOC app. If you were in the POSTHOC app group, we will ask you about your experiences with

the app. This interview will take 15-30 minutes and we will record the audio of the conversation (not video) if that is okay with you.

All of these research activities can be done remotely. However, you are welcome to come in and we will help you download the app onto your device, show you how to use the app, complete questionnaires, and/or complete other study activities. We are located in the School of Nursing, across the street from Greenebaum Comprehensive Cancer Center.

WHAT ARE MY RESPONSIBILITIES IF I TAKE PART IN THIS RESEARCH?

If you take part in this research, you will be responsible for participating in the study activities, as outlined above.

POTENTIAL RISKS/DISCOMFORTS:

As with all research studies, there are risks associated with the study activities. We have taken measures to minimize all anticipated risks. Please consider these risks while deciding if you want to participate.

1. *Emotional distress*
   You could become upset or overwhelmed by the expectation to answer questions about your cancer experience and cancer-related symptoms. However, we want to emphasize that you are not "in trouble" if you do not follow the procedures exactly. We want to use what we learn from this study to improve the procedures for the next study.

   Our questionnaires contain information that might be distressing or private (e.g., "I am satisfied with family communication about my illness"). You do not have to answer any questions you are not comfortable answering, and you can take a break or stop answering the questionnaires at any time.

2. *Breach of confidentiality*
   There is always a risk of a breach of confidentiality in which sensitive medical information could become known to people outside the research team. To avoid leakage of sensitive information, only Dr. Kleckner (the study chair), the study coordinator(s), and any individual designees will have access to the screening log and the file that links your name with your subject number (both will be encrypted); these files will be stored on password-protected computers in their private offices. All data files will reference you by a non-identifiable Participant ID and will be stored on Dr. Kleckner's computer and secure servers at UMB. All consent forms will be stored in a locked cabinet also in her or her staff's office. All audio-recorded interviews will be transferred to Dr. Kleckner's secure computer and server at UMB within 2 business days of the interview and then immediately deleted from the recorder. All interview file names will not include your name or any identifying information. If Dr. Kleckner shares data with any other researcher for analyses, all data will be de-identified (i.e., will not have your name, birthdate, contact information, etc.). Presentation of study findings in the form of talks and manuscripts, either in private or public settings, will not have any identifiable information, nor will any audio clips ever be played in public. Dr. Kleckner and all other co-investigators participate in ethical training in accordance with institutional policies.

HP-00100473 UM IRB Approval Date 5/23/2023

All data from the Fitbit will be continuously and passively streamed to the Fitbit servers. Other data collected from the study, including on the POSTHOC app, will not be shared with Fitbit.

POTENTIAL BENEFITS

You might or might not benefit from being in this research study. You may become more aware of your diet, physical activity, and sleep patterns, which can contribute to overall health. You may also feel a sense of satisfaction from contributing to research that is designed to help patients in the future.

ALTERNATIVES TO PARTICIPATION

This is not a treatment study, and the alternative is to not participate. Instead of participating, you may choose to log your diet, physical activity, and sleep patterns on your own, and/or see a clinician (e.g., dietitian, primary care physician) to explore how your diet, physical activity, and sleep patterns may be contributing to your overall symptoms.

CONFIDENTIALITY AND ACCESS TO RECORDS

Using your medical record number, we will access your electronic medical record so that we can get information regarding your cancer diagnosis, cancer treatment history, medical history, and social history. We will collect your name, address, phone number, and email address in order to contact you for scheduling and reminders of upcoming study activities. Only Dr. Amber Kleckner, the principal investigator, and her trained and designated research personnel will have access to confidential information. All confidential information that includes personally identifiable information will be coded with a study ID number. The principal investigator and study coordinator(s) will be the only individuals with access to the key of the assigned ID numbers. All confidential information will be locked in a cabinet in a secured location at the University of Maryland, School of Nursing. Your personally identifiable information will not be used for this study's analyses, but it will be kept on file if federal agencies or the Institutional Review Board (IRB) are mandated to review any information.

All study records will be considered confidential, and all participants' names and personally identifiable information will not be used in reports or publications. Efforts will be made to limit access to your personal information, including research study records, to people involved with the study who have a need to review this information. We cannot promise complete secrecy. Entities that may inspect and copy your information include the IRB and other representatives of this organization. Those designated from the University of Maryland will be allowed to examine certain research records of this study; however, anyone inspecting this information is required to keep this personal information confidential. Your personal information will not be released unless mandated by law. By signing this document, you are authorizing this access to the monitors, auditors, and the IRB.

All de-identified data from federally funded studies are required to be made publicly available, effective January 25, 2023. The majority of this study was federally funded before that date. However, deidentified transcripts of the exit interviews may be made posted in a public database.

The data from the study may be published. However, you will not be identified. People designated from University of Maryland and people from the sponsor will be allowed to inspect sections of your medical

and research records related to the study. Everyone using study information will work to keep your personal information confidential. Your personal information will not be given out unless required by law.

A description of this clinical trial is available on https://www.clinicaltrials.gov, identifier NCT05499663, as required by U. S. Law. This website will not include information that can identify you. At most, the website will include a summary of the results. You can search this website at any time.

We are using the POSTHOC app to deliver a digital version of your Survivorship Care Plan. In addition, we are using the app to collect data regarding the severity of four common symptoms – distress, fatigue, pain, numbness/tingling, and how much these symptoms are interfering with your daily activities. The POSTHOC app was developed by researchers at Charles River Analytics in Cambridge, Massachusetts. However, all your data from the app will be stored locally at UMB. **Charles River Analytics will not be able to view any personally identifiable information (e.g., name) or medical information (e.g., details of your cancer diagnosis) or any details that you or we input into the app.**

RIGHT TO WITHDRAW

Your participation in this study is voluntary. You do not have to take part in this research. You are free to withdraw your consent at any time. Refusal to take part or to stop taking part in the study will involve no penalty or loss of benefits to which you are otherwise entitled. If you have questions, concerns, or complaints, or if you need to report a medical injury related to the research, please contact the investigator, Dr. Amber Kleckner, at 410-706-5961. To discontinue your participation in the study, a written withdrawal is requested, sent to Dr. Amber Kleckner at amber.kleckner@umaryland.edu.

If you withdraw from this study, already collected data will not be removed from the study database. You will be asked whether the investigator can collect data from your routine medical care. If you agree, these data will be handled the same as research data.

You will be told of any significant new findings that develop during the study that may affect your willingness to continue participation.

CAN I BE REMOVED FROM THE RESEARCH?

You may be withdrawn from the study if your health becomes worse or if your doctor feels that staying in the study is harmful to your health. The study team also holds the discretion to withdraw you from the study for any reason. For example, if you do not complete the first questionnaire and attempt to use the app within 3 weeks of consenting to the study, we will remove you from the study

The sponsor can also end the research study early. The study chair will tell you about this and you will have the chance to ask questions if this were to happen.

COSTS TO PARTICIPANTS

There will be no fee to enroll in the study. However, you or your insurance will be billed for costs of medical care that you would have needed or received if you were not in the study.

HP-00100473 UM IRB Approval Date 5/23/2023

PAYMENT TO PARTICIPANTS

You will be compensated for your time to complete the study activities. We will pay you a total of $50 to complete all study activities. Payment will be delivered at the end of the third assessment (after Week 12) in the form of cash, electronic gift card, or check. All subjects who complete the study can keep the Fitbit activity tracker for free. If you decide to withdraw before completion of the study, your compensation will be prorated.

STUDY-RELATED INJURY

If you have an injury, promptly seek medical care from any healthcare provider. <u>If you have an emergency, call 911 or go to the nearest emergency room.</u> You should tell the healthcare provider that you have participated in a research study.

If you believe the injury is related to the study, notify the PI, Dr. Amber Kleckner, at 410-706-5961. UMB, if requested, will assist you to get medical care or referrals.

UMB and/or its affiliated healthcare facilities or healthcare providers will not provide any financial compensation or reimbursement to you for the cost of medical care or other expenses arising from an injury.

In such cases, you or your insurance may be billed for the costs of medical care.

UNIVERSITY STATEMENT

The University of Maryland, Baltimore (UMB) is committed to providing participants in its research studies all rights due to them under State and federal law. You give up none of your legal rights by signing this consent form or by participating in this study. This study has been reviewed and approved by an Institutional Review Board (IRB). The IRB is a group of scientists, physicians, experts, and community representatives. The IRB's membership includes persons who are not affiliated with UMB and persons who do not conduct research studies.

If you have questions, concerns, complaints, or believe you have been harmed through participation in this study as a result of researcher negligence, you can contact members of the IRB or the Human Research Protections Office (HRPO) to ask questions, discuss problems or concerns, obtain information, or offer input about your rights as a research participant. The contact information for the IRB and the HRPO is:

University of Maryland, Baltimore
Institutional Review Board
Human Research Protections Office
620 W. Lexington Street, Second Floor
Baltimore, MD 21201
410-706-5037

HP-00100473 UM IRB Approval Date 5/23/2023

Signing this consent form indicates that you have read this consent form (or have had it read to you), that your questions have been answered to your satisfaction, and that you voluntarily agree to participate in this research study. You will receive a copy of this signed consent form.

If you agree to participate in this study, please sign your name below.

____
Participant's Signature

Date: _______________________________

____
Investigator or Designee Obtaining Consent Signature

Date: _______________________________

HP-00100473 UM IRB Approval Date 5/23/2023

Health Insurance Portability and Accountability Act (HIPAA)
AUTHORIZATION TO OBTAIN, USE AND DISCLOSE PROTECTED HEALTH INFORMATION FOR RESEARCH

Name of Study Participant: ____

Date of Birth: ________________ **Medical Record Number:** ____________

Name of this Research Study: *Leveraging technology to address health outcomes of cancer survivors*

UMB IRB Approval Number: *HP-00100473*

Researcher's Name: *Amber Kleckner, PhD*

Researcher's Contact Information: *Department of Pain & Translational Symptom Science*
University of Maryland School of Nursing
655 W. Lombard St.
Baltimore, MD 21201
410-706-5961

This research study will use health information that identifies you. If you agree to participate, this researcher will use just the health information listed below.

The specific health information to be used or shared:
- Demographics (e.g., height, weight, age, race, ethnicity, education, marital status)
- Details regarding cancer diagnosis and treatment (e.g., cancer site, cancer stage, chemotherapy type and dosing, surgical procedures, hormone therapy)
- Clinical characteristics (e.g., current menopausal status, Karnofsky Performance Status)
- Most recent blood work (e.g., hemoglobin, hematocrit, lymphocytes, etc.)
- Medical history (e.g., prior myocardial infarction, diabetes status)

Federal laws require this researcher to protect the privacy of this health information. She will share it only with the people and groups described here.

People and organizations who will use or share this information:
- Dr. Amber Kleckner and her research team
- The sponsor of the study, or its agents, such as data repositories or contract research organizations
- Organization that will coordinate health care billing or compliance such as offices within University of Maryland School of Nursing; the University of Maryland, Baltimore (UMB); University Physicians, Inc. (UPI) and the faculty practices of the UMB; and the University of Maryland Medical System (UMMS)

HP-00100473 UM IRB Approval Date 5/23/2023

This Authorization will not expire, but you can revoke it at any time.
To revoke this Authorization, send a letter or email to this researcher stating your decision. She will stop collecting health information about you, and she will not allow you to continue in this study. She can use or share health information already gathered.

Additional information:

- You can refuse to sign this form. If you do not sign it, you cannot participate in this study. This will not affect the care you receive at:
  - University Physicians, Inc. (UPI)
  - University of Maryland Medical System (UMMS)

  It will not cause any loss of benefits to which you are otherwise entitled.
- Sometimes, government agencies such as the Food and Drug Administration or the Department of Social Services request copies of health information. The law may require this researcher, the University of Maryland School of Nursing, UPI, or UMMS to give it to them.
- This researcher will take reasonable steps to protect your health information. However, federal protection laws may not apply to people or groups outside the University of Maryland School of Nursing, UMB, UPI, or UMMS.
- Except for certain special cases, you have the right to a copy of your health information created during this research study. You may have to wait until the study ends. Ask this researcher how to get a copy of this information from her.

My signature indicates that I authorize the use and sharing of my protected health information for the purposes described above. I also permit my doctors and other health care providers to share my protected health information with this researcher for the purposes described above.

Signature: ___________________________________ Date: ______________________

Name (printed) ______________________________________

Privacy Questions? Call the UMSOM Privacy Official (410-706-0337) with questions about your rights and protections under privacy rules.

Other Questions? Call the researcher named on this form with any other questions.

HP-00100473 UM IRB Approval Date 5/23/2023
