# Supplementary material for: Digitizing Survivorship Care Plans Through the POST-Treatment Health Outcomes of Cancer Survivors (POSTHOC) Mobile App: Protocol for a Phase II Randomized Controlled Trial
Source: JMIR Res Protoc. 2024 Sep 5;13:e59222. doi: 10.2196/59222 (PMC11413545; doi:10.2196/59222)
Supplement: Multimedia Appendix 2 [file resprot_v13i1e59222_app2.docx]

# Supplementary Materials

## Informed Consent Process

Participants will have the option of providing consent in-person using the computer (eConsent). For the written consent, a member of the study team will meet with the potential participant in a private room. She or he will go through the consent form with the patient face-to-face and answer any questions they may have. They will then be given the option to sign the consent form then or at a later date. Instead of the paper-based consent document, consent may occur using the IRB-approved eConsent document provided via REDCap. In this case, the study team reviews the consent form thoroughly via phone or videoconference (if they haven’t already) to ensure comprehension. Once the eConsent form is signed and submitted, the patient will be able to receive a printout of the paper copy, download a digital version, and/or receive an email with a digital attachment of the signed form.

We will ensure that participants are aware that consent is a voluntary process and that they can withdraw at any time. Because this study includes only one “phase,” we will not ask participants to re-consent during the study unless there is a change to the study procedures or other pertinent information that would change a person's decision to participate in the study. The consent form will also contain information regarding HIPAA authorization.

## Privacy and Confidentiality

Participants' privacy and confidentiality will be protected in several ways. At the beginning of a session, each participant will be assigned an alphanumeric code that will be written on all their response materials (except for their consent form) and data files instead of their names. This procedure will ensure that participants' identities will never be directly associated with their specific data. To protect participant confidentiality, all paperwork that could directly be linked to the participant will be stored in the investigators' locked filing cabinets. All other data will be coded (i.e., identified by code rather than with personal information) and stored in a locked cabinet or on a computer where a password is required for entry and decryption. The Site PI, Dr. Kleckner, will distribute passwords and keys. Only adequately trained study staff who require data access to complete the study will receive a password or key.

Privacy will be ensured by making appointments with participants to call them or have a video conference about the study. If a participant elects to come in, our clinical suites are located in a discrete laboratory in the School of Nursing.

## **Table S1. Daily Nutrition Questionnaire**

For participants in the intervention group who chose to focus on nutrition for the duration of the study they will be asked to respond to six questions at the end of each day. Their responses will be compared to goals on their digital Survivorship Care Plan, and feedback will be provided to encourage adherence to the Plan.

| Daily Nutrition Questions |
| --- |
| 1. How many servings of fresh, canned, frozen, or dried fruits and/or vegetables did you eat today? 2. How many servings of meat/fish/beans/eggs did you eat today? 3. How many servings of whole grains did you eat today? 4. How many regular soda, sweet tea, energy/sports drinks, sweetened coffee, or other sugar-sweetened beverages did you drink today? 5. How many times today did you eat sweet foods or desserts, such as cake, cookies, pastries, donuts, muffins, and candies? 6. How many alcoholic beverages did you consume today? |
