# Supplementary material for: Digitizing Survivorship Care Plans Through the POST-Treatment Health Outcomes of Cancer Survivors (POSTHOC) Mobile App: Protocol for a Phase II Randomized Controlled Trial
Source: JMIR Res Protoc. 2024 Sep 5;13:e59222. doi: 10.2196/59222 (PMC11413545; doi:10.2196/59222)
Supplement: Multimedia Appendix 3 [file resprot_v13i1e59222_app3.pdf]

CONSORT-EHEALTH (V 1.6.1) - Submission/Publication Form

The CONSORT-EHEALTH checklist is intended for authors of randomized trials evaluating web-based and Internet-based applications/interventions, including mobile interventions, electronic games (incl multiplayer games), social media, certain telehealth applications, and other interactive and/or networked electronic applications. Some of the items (e.g. all subitems under item 5 - description of the intervention) may also be applicable for other study designs.

The goal of the CONSORT EHEALTH checklist and guideline is to be
a) a guide for reporting for authors of RCTs,
b) to form a basis for appraisal of an ehealth trial (in terms of validity)

CONSORT-EHEALTH items/subitems are MANDATORY reporting items for studies published in the Journal of Medical Internet Research and other journals / scientific societies endorsing the checklist.

Items numbered 1., 2., 3., 4a., 4b etc are original CONSORT or CONSORT-NPT (non-pharmacologic treatment) items.
Items with Roman numerals (i., ii, iii, iv etc.) are CONSORT-EHEALTH extensions/clarifications.

As the CONSORT-EHEALTH checklist is still considered in a formative stage, we would ask that you also RATE ON A SCALE OF 1-5 how important/useful you feel each item is FOR THE PURPOSE OF THE CHECKLIST and reporting guideline (optional).

Mandatory reporting items are marked with a red *.
In the textboxes, either copy & paste the relevant sections from your manuscript into this form - please include any quotes from your manuscript in QUOTATION MARKS,
or answer directly by providing additional information not in the manuscript, or elaborating on why the item was not relevant for this study.

YOUR ANSWERS WILL BE PUBLISHED AS A SUPPLEMENTARY FILE TO YOUR PUBLICATION IN JMIR AND ARE CONSIDERED PART OF YOUR PUBLICATION (IF ACCEPTED).
Please fill in these questions diligently. Information will not be copyedited, so please use proper spelling and grammar, use correct capitalization, and avoid abbreviations.

DO NOT FORGET TO SAVE AS PDF _AND_ CLICK THE SUBMIT BUTTON SO YOUR ANSWERS ARE IN OUR DATABASE !!!

Citation Suggestion (if you append the pdf as Appendix we suggest to cite this paper in the caption):
Eysenbach G, CONSORT-EHEALTH Group

CONSORT-EHEALTH: Improving and Standardizing Evaluation Reports of Web-based and Mobile Health Interventions
J Med Internet Res 2011;13(4):e126
URL: http://www.jmir.org/2011/4/e126/
doi: 10.2196/jmir.1923
PMID: 22209829

amber.kleckner@gmail.com Switch account

Not shared

Draft saved

* Indicates required question

Your name *

First Last

Amber Kleckner

Primary Affiliation (short), City, Country *

University of Toronto, Toronto, Canada

University of Maryland Baltimore, Baltimore, Ur

Your e-mail address *

abc@gmail.com

amber.kleckner@umaryland.edu

Title of your manuscript *

Provide the (draft) title of your manuscript.

Leveraging Health Technology to Address POST-Treatment Health Outcomes of Cancer Survivors (POSTHOC): Protocol for a Phase II Survivorship Care Plan Mobile App Randomized Controlled Trial

Name of your App/Software/Intervention *

If there is a short and a long/alternate name, write the short name first and add the long name in brackets.

POSTHOC

Evaluated Version (if any)

e.g. "V1", "Release 2017-03-01", "Version 2.0.27913"

v1.1.11 - v1.1.13

Language(s) *

What language is the intervention/app in? If multiple languages are available, separate by comma (e.g. "English, French")

English

URL of your Intervention Website or App

e.g. a direct link to the mobile app on app in appstore (itunes, Google Play), or URL of the website. If the intervention is a DVD or hardware, you can also link to an Amazon page.

https://play.google.com/apps/test/com.cra.sparkcare.posthoc/39

URL of an image/screenshot (optional)

Your answer

Accessibility *

Can an enduser access the intervention presently?

- ○ access is free and open
- ◉ access only for special usergroups, not open
- ○ access is open to everyone, but requires payment/subscription/in-app purchases
- ○ app/intervention no longer accessible
- ○ Other:

Primary Medical Indication/Disease/Condition *

e.g. "Stress", "Diabetes", or define the target group in brackets after the condition, e.g. "Autism (Parents of children with)", "Alzheimers (Informal Caregivers of)"

Cancer

Primary Outcomes measured in trial *

comma-separated list of primary outcomes reported in the trial

feasibility, acceptability, usability

Secondary/other outcomes

Are there any other outcomes the intervention is expected to affect?

cumulative symptom burden

Recommended "Dose" *

What do the instructions for users say on how often the app should be used?

- (x) Approximately Daily
- ( ) Approximately Weekly
- ( ) Approximately Monthly
- ( ) Approximately Yearly
- ( ) "as needed"
- ( ) Other:

Approx. Percentage of Users (starters) still using the app as recommended after 3 months *

- [x] unknown / not evaluated
- [ ] 0-10%
- [ ] 11-20%
- [ ] 21-30%
- [ ] 31-40%
- [ ] 41-50%
- [ ] 51-60%
- [ ] 61-70%
- [ ] 71%-80%
- [ ] 81-90%
- [ ] 91-100%
- [ ] Other:

Overall, was the app/intervention effective? *

- [ ] yes: all primary outcomes were significantly better in intervention group vs control
- [ ] partly: SOME primary outcomes were significantly better in intervention group vs control
- [ ] no statistically significant difference between control and intervention
- [ ] potentially harmful: control was significantly better than intervention in one or more outcomes
- [ ] inconclusive: more research is needed
- [x] Other: N/A This is a protocol paper and the work is not yet done

Article Preparation Status/Stage *

At which stage in your article preparation are you currently (at the time you fill in this form)

- ○ not submitted yet - in early draft status
- ○ not submitted yet - in late draft status, just before submission
- ○ submitted to a journal but not reviewed yet
- ◉ submitted to a journal and after receiving initial reviewer comments
- ○ submitted to a journal and accepted, but not published yet
- ○ published
- ○ Other:

Journal *

If you already know where you will submit this paper (or if it is already submitted), please provide the journal name (if it is not JMIR, provide the journal name under "other")

- ○ not submitted yet / unclear where I will submit this
- ○ Journal of Medical Internet Research (JMIR)
- ○ JMIR mHealth and UHealth
- ○ JMIR Serious Games
- ○ JMIR Mental Health
- ○ JMIR Public Health
- ○ JMIR Formative Research
- ◉ Other JMIR sister journal
- ○ Other:

Is this a full powered effectiveness trial or a pilot/feasibility trial? *

- (x) Pilot/feasibility
- ( ) Fully powered

Manuscript tracking number *

If this is a JMIR submission, please provide the manuscript tracking number under "other" (The ms tracking number can be found in the submission acknowledgement email, or when you login as author in JMIR. If the paper is already published in JMIR, then the ms tracking number is the four-digit number at the end of the DOI, to be found at the bottom of each published article in JMIR)

- ( ) no ms number (yet) / not (yet) submitted to / published in JMIR
- (x) Other: 59222

TITLE AND ABSTRACT

1a) TITLE: Identification as a randomized trial in the title

1a) Does your paper address CONSORT item 1a? *

I.e does the title contain the phrase "Randomized Controlled Trial"? (if not, explain the reason under "other")

- (x) yes
- ( ) Other:

1a-i) Identify the mode of delivery in the title

Identify the mode of delivery. Preferably use “web-based” and/or “mobile” and/or “electronic game” in the title. Avoid ambiguous terms like “online”, “virtual”, “interactive”. Use “Internet-based” only if Intervention includes non-web-based Internet components (e.g. email), use “computer-based” or “electronic” only if offline products are used. Use “virtual” only in the context of “virtual reality” (3-D worlds). Use “online” only in the context of “online support groups”. Complement or substitute product names with broader terms for the class of products (such as “mobile” or “smart phone” instead of “iphone”), especially if the application runs on different platforms.

	1	2	3	4	5	
subitem not at all important	○	○	○	○	◉	essential

Clear selection

Does your paper address subitem 1a-i? *

Copy and paste relevant sections from manuscript title (include quotes in quotation marks "like this" to indicate direct quotes from your manuscript), or elaborate on this item by providing additional information not in the ms, or briefly explain why the item is not applicable/relevant for your study

"mobile app" <This answer must contain at least 25 characters but I already answered the question>

1a-ii) Non-web-based components or important co-interventions in title

Mention non-web-based components or important co-interventions in title, if any (e.g., “with telephone support”).

	1	2	3	4	5	
subitem not at all important	○	○	○	○	◉	essential

Clear selection

Does your paper address subitem 1a-ii?

Copy and paste relevant sections from manuscript title (include quotes in quotation marks "like this" to indicate direct quotes from your manuscript), or elaborate on this item by providing additional information not in the ms, or briefly explain why the item is not applicable/relevant for your study

"Survivorship Care Plan" The Survivorship Care Plan is a key non-web-based component.

1a-iii) Primary condition or target group in the title

Mention primary condition or target group in the title, if any (e.g., “for children with Type I Diabetes”) Example: A Web-based and Mobile Intervention with Telephone Support for Children with Type I Diabetes: Randomized Controlled Trial

	1	2	3	4	5	
subitem not at all important	○	○	○	○	◉	essential

Clear selection

Does your paper address subitem 1a-iii? *

Copy and paste relevant sections from manuscript title (include quotes in quotation marks "like this" to indicate direct quotes from your manuscript), or elaborate on this item by providing additional information not in the ms, or briefly explain why the item is not applicable/relevant for your study

"Cancer Survivors" <extra characters to meet the 25-character minimum>

1b) ABSTRACT: Structured summary of trial design, methods, results, and conclusions

NPT extension: Description of experimental treatment, comparator, care providers, centers, and blinding status.

1b-i) Key features/functionalities/components of the intervention and comparator in the METHODS section of the ABSTRACT

Mention key features/functionalities/components of the intervention and comparator in the abstract. If possible, also mention theories and principles used for designing the site. Keep in mind the needs of systematic reviewers and indexers by including important synonyms. (Note: Only report in the abstract what the main paper is reporting. If this information is missing from the main body of text, consider adding it)

	1	2	3	4	5	
subitem not at all important	○	○	○	○	◉	essential

Clear selection

Does your paper address subitem 1b-i? *

Copy and paste relevant sections from the manuscript abstract (include quotes in quotation marks "like this" to indicate direct quotes from your manuscript), or elaborate on this item by providing additional information not in the ms, or briefly explain why the item is not applicable/relevant for your study

"Those randomized to the POSTHOC group will be provided with their survivorship care plan via the app and will choose to focus on nutrition or exercise for the duration of the study, based on their individual plan and personal preferences. Those randomized to the control group will get a paper-based plan."

1b-ii) Level of human involvement in the METHODS section of the ABSTRACT

Clarify the level of human involvement in the abstract, e.g., use phrases like “fully automated” vs. “therapist/nurse/care provider/physician-assisted” (mention number and expertise of providers involved, if any). (Note: Only report in the abstract what the main paper is reporting. If this information is missing from the main body of text, consider adding it)

	1	2	3	4	5	
subitem not at all important	○	◉	○	○	○	essential

Clear selection

Does your paper address subitem 1b-ii?

Copy and paste relevant sections from the manuscript abstract (include quotes in quotation marks "like this" to indicate direct quotes from your manuscript), or elaborate on this item by providing additional information not in the ms, or briefly explain why the item is not applicable/relevant for your study

This app currently has data that is entered into it by a human, with plans for it to automatically be linked to medical records. The level of human involvement at this stage is not relevant to the participant experience.

1b-iii) Open vs. closed, web-based (self-assessment) vs. face-to-face assessments in the METHODS section of the ABSTRACT

Mention how participants were recruited (online vs. offline), e.g., from an open access website or from a clinic or a closed online user group (closed usergroup trial), and clarify if this was a purely web-based trial, or there were face-to-face components (as part of the intervention or for assessment). Clearly say if outcomes were self-assessed through questionnaires (as common in web-based trials). Note: In traditional offline trials, an open trial (open-label trial) is a type of clinical trial in which both the researchers and participants know which treatment is being administered. To avoid confusion, use “blinded” or “unblinded” to indicated the level of blinding instead of “open”, as “open” in web-based trials usually refers to “open access” (i.e. participants can self-enrol). (Note: Only report in the abstract what the main paper is reporting. If this information is missing from the main body of text, consider adding it)

	1	2	3	4	5	
subitem not at all important	○	◉	○	○	○	essential

Clear selection

Does your paper address subitem 1b-iii?

Copy and paste relevant sections from the manuscript abstract (include quotes in quotation marks "like this" to indicate direct quotes from your manuscript), or elaborate on this item by providing additional information not in the ms, or briefly explain why the item is not applicable/relevant for your study

"We will recruit 54 patients who have recently completed curative therapy for cancer (any type) in-person and remotely. They will be randomized 2:1, POSTHOC:usual care (unblinded)... At baseline, 6 weeks, and 12 weeks, we will evaluate patient-reported outcomes including total symptom burden (online questionnaire)..."

1b-iv) RESULTS section in abstract must contain use data

Report number of participants enrolled/assessed in each group, the use/uptake of the intervention (e.g., attrition/adherence metrics, use over time, number of logins etc.), in addition to primary/secondary outcomes. (Note: Only report in the abstract what the main paper is reporting. If this information is missing from the main body of text, consider adding it)

	1	2	3	4	5	
subitem not at all important	○	●	○	○	○	essential

Clear selection

Does your paper address subitem 1b-iv?

Copy and paste relevant sections from the manuscript abstract (include quotes in quotation marks "like this" to indicate direct quotes from your manuscript), or elaborate on this item by providing additional information not in the ms, or briefly explain why the item is not applicable/relevant for your study

"This trial is open to accrual in the University of Maryland Medical System as of March 2024 and as of July 2024, there are 18 participants enrolled."

1b-v) CONCLUSIONS/DISCUSSION in abstract for negative trials

Conclusions/Discussions in abstract for negative trials: Discuss the primary outcome - if the trial is negative (primary outcome not changed), and the intervention was not used, discuss whether negative results are attributable to lack of uptake and discuss reasons. (Note: Only report in the abstract what the main paper is reporting. If this information is missing from the main body of text, consider adding it)

	1	2	3	4	5	
subitem not at all important	○	◉	○	○	○	essential

Clear selection

Does your paper address subitem 1b-v?

Copy and paste relevant sections from the manuscript abstract (include quotes in quotation marks "like this" to indicate direct quotes from your manuscript), or elaborate on this item by providing additional information not in the ms, or briefly explain why the item is not applicable/relevant for your study

This is a protocol paper: "Our results will provide evidence about the effects of self-management of health on healthcare usage and treatment symptoms. This knowledge will be integral to larger randomized controlled studies, integration with the electronic medical record, and nationwide implementation."

INTRODUCTION

2a) In INTRODUCTION: Scientific background and explanation of rationale

2a-i) Problem and the type of system/solution

Describe the problem and the type of system/solution that is object of the study: intended as stand-alone intervention vs. incorporated in broader health care program? Intended for a particular patient population? Goals of the intervention, e.g., being more cost-effective to other interventions, replace or complement other solutions? (Note: Details about the intervention are provided in “Methods” under 5)

	1	2	3	4	5	
subitem not at all important	○	○	○	○	◉	essential

Clear selection

Does your paper address subitem 2a-i? *

Copy and paste relevant sections from the manuscript (include quotes in quotation marks "like this" to indicate direct quotes from your manuscript), or elaborate on this item by providing additional information not in the ms, or briefly explain why the item is not applicable/relevant for your study

"Additional tools are needed to supplement the [Survivorship Care Plan] to promote self-management, encourage achievement and/or maintenance of healthy lifestyle behaviors, and facilitate the detection of risk for post-treatment complications."

2a-ii) Scientific background, rationale: What is known about the (type of) system

Scientific background, rationale: What is known about the (type of) system that is the object of the study (be sure to discuss the use of similar systems for other conditions/diagnoses, if appropiate), motivation for the study, i.e. what are the reasons for and what is the context for this specific study, from which stakeholder viewpoint is the study performed, potential impact of findings [2]. Briefly justify the choice of the comparator.

	1	2	3	4	5	
subitem not at all important	○	○	○	○	◉	essential

Clear selection

Does your paper address subitem 2a-ii? *

Copy and paste relevant sections from the manuscript (include quotes in quotation marks "like this" to indicate direct quotes from your manuscript), or elaborate on this item by providing additional information not in the ms, or briefly explain why the item is not applicable/relevant for your study

"One way to address issues with current SCP practices is through mobile health (mHealth) interventions. mHealth data capabilities have grown significantly due to greater adoption of smart devices, improved mobile network coverage, and increased focus of American culture on self-monitoring and preventative health [9]. "

2b) In INTRODUCTION: Specific objectives or hypotheses

Does your paper address CONSORT subitem 2b? *

Copy and paste relevant sections from the manuscript (include quotes in quotation marks "like this" to indicate direct quotes from your manuscript), or elaborate on this item by providing additional information not in the ms, or briefly explain why the item is not applicable/relevant for your study

"This study will fill a significant gap in the literature by being the first study, to our knowledge, to both digitize the SCP in a mobile application integrated with a wearable activity tracker and test the effects of the app on symptom burden in cancer survivors. We hypothesize that 1) the POSTHOC app is feasible, acceptable, and useful for cancer survivors, 2) the POSTHOC app will reduce cumulative symptom burden reported by patients compared to usual care, and 3) the POSTHOC app will improve health behaviors compared to usual care, as measured by patient-reported physical activity. "

METHODS

3a) Description of trial design (such as parallel, factorial) including allocation ratio

Does your paper address CONSORT subitem 3a? *

Copy and paste relevant sections from the manuscript (include quotes in quotation marks "like this" to indicate direct quotes from your manuscript), or elaborate on this item by providing additional information not in the ms, or briefly explain why the item is not applicable/relevant for your study

"This phase II feasibility/preliminary efficacy parallel-arm randomized controlled trial..."
"...participants will be randomized 2:1 for POSTHOC app (n=36) vs. control (n=18)..."

3b) Important changes to methods after trial commencement (such as eligibility criteria), with reasons

Does your paper address CONSORT subitem 3b? *

Copy and paste relevant sections from the manuscript (include quotes in quotation marks "like this" to indicate direct quotes from your manuscript), or elaborate on this item by providing additional information not in the ms, or briefly explain why the item is not applicable/relevant for your study

No changes have been made to eligibility after trial commencement.

3b-i) Bug fixes, Downtimes, Content Changes

Bug fixes, Downtimes, Content Changes: ehealth systems are often dynamic systems. A description of changes to methods therefore also includes important changes made on the intervention or comparator during the trial (e.g., major bug fixes or changes in the functionality or content) (5-iii) and other “unexpected events” that may have influenced study design such as staff changes, system failures/downtimes, etc. [2].

	1	2	3	4	5	
subitem not at all important	○	○	●	○	○	essential

Clear selection

Does your paper address subitem 3b-i?

Copy and paste relevant sections from the manuscript (include quotes in quotation marks "like this" to indicate direct quotes from your manuscript), or elaborate on this item by providing additional information not in the ms, or briefly explain why the item is not applicable/relevant for your study

There have not yet been major changes to the functionality or content, etc. that we need to describe.

4a) Eligibility criteria for participants

Does your paper address CONSORT subitem 4a? *

Copy and paste relevant sections from the manuscript (include quotes in quotation marks "like this" to indicate direct quotes from your manuscript), or elaborate on this item by providing additional information not in the ms, or briefly explain why the item is not applicable/relevant for your study

"To be eligible for the study, patients must 1) be ≥18 years, 2) have had a cancer diagnosis (any type), 3) will soon or have recently completed treatment (within the past 12 weeks) with chemotherapy, radiotherapy, or surgery with curative intent, 4) have received, plans to receive, or is open to receiving a Survivorship Care Plan as per their provider, 5) have access to a device capable of running the POSTHOC app and Fitbit app (e.g., Android or Apple smartphone) and reliable Internet access, 6) be able to read and understand English, and 7) be able to provide written informed consent. Patients must not have planned surgery, radiotherapy, or chemotherapy during the study period (hormonal and biologic therapy is allowed). "

4a-i) Computer / Internet literacy

Computer / Internet literacy is often an implicit “de facto” eligibility criterion - this should be explicitly clarified.

	1	2	3	4	5	
subitem not at all important	○	○	●	○	○	essential

Clear selection

Does your paper address subitem 4a-i?

Copy and paste relevant sections from the manuscript (include quotes in quotation marks "like this" to indicate direct quotes from your manuscript), or elaborate on this item by providing additional information not in the ms, or briefly explain why the item is not applicable/relevant for your study

"Digital literacy is an implicit “de facto” eligibility criterion."

4a-ii) Open vs. closed, web-based vs. face-to-face assessments:

Open vs. closed, web-based vs. face-to-face assessments: Mention how participants were recruited (online vs. offline), e.g., from an open access website or from a clinic, and clarify if this was a purely web-based trial, or there were face-to-face components (as part of the intervention or for assessment), i.e., to what degree got the study team to know the participant. In online-only trials, clarify if participants were quasi-anonymous and whether having multiple identities was possible or whether technical or logistical measures (e.g., cookies, email confirmation, phone calls) were used to detect/prevent these.

	1	2	3	4	5	
subitem not at all important	○	○	○	●	○	essential

Clear selection

Does your paper address subitem 4a-ii? *

Copy and paste relevant sections from the manuscript (include quotes in quotation marks "like this" to indicate direct quotes from your manuscript), or elaborate on this item by providing additional information not in the ms, or briefly explain why the item is not applicable/relevant for your study

"Potential participants will be identified using four methods: a) screening medical records, b) direct referral from providers (e.g., nurses, nurse navigators, physicians), c) flyers, and d) advertising via standard UMB networks (e.g., ResearchMatch). After a potential participant has shown an initial interest in the study, the study team will review eligibility and discuss the study activities. If the person volunteers to participate, study personnel can obtain informed consent in-person or remotely via phone or videoconference and electronic remote consent. "

"All study activities can be done remotely and there is no essential face-to-face interaction between the study team and the participant. "

4a-iii) Information giving during recruitment

Information given during recruitment. Specify how participants were briefed for recruitment and in the informed consent procedures (e.g., publish the informed consent documentation as appendix, see also item X26), as this information may have an effect on user self-selection, user expectation and may also bias results.

	1	2	3	4	5	
subitem not at all important	○	○	●	○	○	essential

Clear selection

Does your paper address subitem 4a-iii?

Copy and paste relevant sections from the manuscript (include quotes in quotation marks "like this" to indicate direct quotes from your manuscript), or elaborate on this item by providing additional information not in the ms, or briefly explain why the item is not applicable/relevant for your study

"After a potential participant has shown an initial interest in the study, the study team will review eligibility and the consent form, which includes a and discuss detailed discussion of the study activities (see Appendix for a copy of the consent form). "

4b) Settings and locations where the data were collected

Does your paper address CONSORT subitem 4b? *

Copy and paste relevant sections from the manuscript (include quotes in quotation marks "like this" to indicate direct quotes from your manuscript), or elaborate on this item by providing additional information not in the ms, or briefly explain why the item is not applicable/relevant for your study

"Recruitment will occur from University of Maryland Medical System (UMMS) cancer centers including the University of Maryland Greenebaum Comprehensive Cancer Center (UMGCCC) in Baltimore, MD; the Cancer Center at UM Shore Regional Health in Easton, MD; the Cancer Institute at UM St. Joseph Medical Center in Towson, MD; Kaufman Cancer Center at UM Upper Chesapeake Health in Bel Air, MD; and the Tate Cancer Center at UM Baltimore Washington Medical Center in Glen Burnie, MD."

"Potential participants will be identified using four methods: a) screening medical records, b) direct referral from providers (e.g., nurses, nurse navigators, physicians), c) flyers, and d) advertising via standard UMB networks (e.g., ResearchMatch). "

4b-i) Report if outcomes were (self-)assessed through online questionnaires

Clearly report if outcomes were (self-)assessed through online questionnaires (as common in web-based trials) or otherwise.

	1	2	3	4	5	
subitem not at all important	○	○	○	○	◉	essential

Clear selection

Does your paper address subitem 4b-i? *

Copy and paste relevant sections from the manuscript (include quotes in quotation marks "like this" to indicate direct quotes from your manuscript), or elaborate on this item by providing additional information not in the ms, or briefly explain why the item is not applicable/relevant for your study

"Physical activity and sleep will be measured via a Fitbit Charge 6, which will be given at no cost to the participants. They will be asked to wear the Fitbit for 7 days during week 0 (baseline), week 6, and week 12.
Dietary intake will be assessed using the ASA24® Dietary Assessment Tool [29]. The ASA24® is a freely available web-based tool used for epidemiologic, interventional, behavioral, or clinical research that enables multiple automatically coded 24-hour dietary recalls. It will be administered by a licensed dietitian or nurse via telephone or video conference and takes approximately 30-45 minutes.
EMA will be assessed via the POSTHOC app four times throughout the day for seven days at weeks 0, 6, and 12. The app will alert the participant to report the severity of four common sy4mptoms—pain, distress, fatigue, numbness/tingling, and interference of symptoms with daily activity on a scale of 0-10. While research studies have shown success with this type of data collection [32], participants will have the option to “turn off notifications” if it is too burdensome."

"Questionnaires will be administered via REDCap [33] or on paper and then manually entered into REDCap at weeks 0, 6, and 12. There are 14 questionnaires; 12 of them are self-administered and the remaining two are researcher-administered. "

4b-ii) Report how institutional affiliations are displayed

Report how institutional affiliations are displayed to potential participants [on ehealth media], as affiliations with prestigious hospitals or universities may affect volunteer rates, use, and reactions with regards to an intervention.(Not a required item – describe only if this may bias results)

	1	2	3	4	5	
subitem not at all important	○	◉	○	○	○	essential

Clear selection

Does your paper address subitem 4b-ii?

Copy and paste relevant sections from the manuscript (include quotes in quotation marks "like this" to indicate direct quotes from your manuscript), or elaborate on this item by providing additional information not in the ms, or briefly explain why the item is not applicable/relevant for your study

Our consent form is uploaded as part of the Appendix and contains the University of Maryland logo and information. It is made clear the the participants that this study is being conducted by researchers at the University of Maryland during the consent process.

5) The interventions for each group with sufficient details to allow replication, including how and when they were actually administered

5-i) Mention names, credential, affiliations of the developers, sponsors, and owners

Mention names, credential, affiliations of the developers, sponsors, and owners [6] (if authors/evaluators are owners or developer of the software, this needs to be declared in a "Conflict of interest" section or mentioned elsewhere in the manuscript).

	1	2	3	4	5	
subitem not at all important	○	○	○	○	◉	essential

Clear selection

Does your paper address subitem 5-i?

Copy and paste relevant sections from the manuscript (include quotes in quotation marks "like this" to indicate direct quotes from your manuscript), or elaborate on this item by providing additional information not in the ms, or briefly explain why the item is not applicable/relevant for your study

"The POSTHOC application ("app") was designed by Charles River Analytics specifically for this project"

"Conflicts of Interest. ATW and NMM are employed by Charles River Analytics. Other authors have no conflicts of interest."

5-ii) Describe the history/development process

Describe the history/development process of the application and previous formative evaluations (e.g., focus groups, usability testing), as these will have an impact on adoption/use rates and help with interpreting results.

	1	2	3	4	5	
subitem not at all important	○	○	○	◉	○	essential

Clear selection

Does your paper address subitem 5-ii?

Copy and paste relevant sections from the manuscript (include quotes in quotation marks "like this" to indicate direct quotes from your manuscript), or elaborate on this item by providing additional information not in the ms, or briefly explain why the item is not applicable/relevant for your study

"Charles River Analytics completed a Phase I project to launch the development of an app to digitize the survivorship care plan, called POSTHOC. Specifically, the team conducted targeted interviews with survivors, clinicians, and researchers to understand how providers track symptoms of cancer with their current tools, including the SCP, and what features could be added to the existing tools and workflows to streamline care. Accordingly, the POSTHOC app was designed to go beyond routine plans to deliver an updatable SCP, collect data regarding ongoing health status, and promote healthy lifestyle behaviors. The success of Phase I of the project enabled us to build upon the platform in Phase II: testing of the app in a clinical population regarding feasibility, usability, and preliminary efficacy to reduce symptom burden. "

5-iii) Revisions and updating

Revisions and updating. Clearly mention the date and/or version number of the application/intervention (and comparator, if applicable) evaluated, or describe whether the intervention underwent major changes during the evaluation process, or whether the development and/or content was “frozen” during the trial. Describe dynamic components such as news feeds or changing content which may have an impact on the replicability of the intervention (for unexpected events see item 3b).

	1	2	3	4	5	
subitem not at all important	○	○	○	●	○	essential

Clear selection

Does your paper address subitem 5-iii?

Copy and paste relevant sections from the manuscript (include quotes in quotation marks "like this" to indicate direct quotes from your manuscript), or elaborate on this item by providing additional information not in the ms, or briefly explain why the item is not applicable/relevant for your study

"The trial opened with v.1.1.11 and as of July 2024 the current version is 1.1.13. Updates were made to increase the speed at which the app syncs with Fitbit and opens for the user."

5-iv) Quality assurance methods

Provide information on quality assurance methods to ensure accuracy and quality of information provided [1], if applicable.

	1	2	3	4	5	
subitem not at all important	○	○	○	○	◉	essential

Clear selection

Does your paper address subitem 5-iv?

Copy and paste relevant sections from the manuscript (include quotes in quotation marks "like this" to indicate direct quotes from your manuscript), or elaborate on this item by providing additional information not in the ms, or briefly explain why the item is not applicable/relevant for your study

"The interventionalists will be trained and will use a checklist to ensure fidelity and prevent drift. "

5-v) Ensure replicability by publishing the source code, and/or providing screenshots/screen-capture video, and/or providing flowcharts of the algorithms used

Ensure replicability by publishing the source code, and/or providing screenshots/screen-capture video, and/or providing flowcharts of the algorithms used. Replicability (i.e., other researchers should in principle be able to replicate the study) is a hallmark of scientific reporting.

	1	2	3	4	5	
subitem not at all important	○	◉	○	○	○	essential

Clear selection

Does your paper address subitem 5-v?

Copy and paste relevant sections from the manuscript (include quotes in quotation marks "like this" to indicate direct quotes from your manuscript), or elaborate on this item by providing additional information not in the ms, or briefly explain why the item is not applicable/relevant for your study

The source code of the app is confidential, though we would be willing to share the app with other researchers wishing to build upon and/or replicate this study.

5-vi) Digital preservation

Digital preservation: Provide the URL of the application, but as the intervention is likely to change or disappear over the course of the years; also make sure the intervention is archived (Internet Archive, webcitation.org, and/or publishing the source code or screenshots/videos alongside the article). As pages behind login screens cannot be archived, consider creating demo pages which are accessible without login.

	1	2	3	4	5	
subitem not at all important	◉	○	○	○	○	essential

Clear selection

Does your paper address subitem 5-vi?

Copy and paste relevant sections from the manuscript (include quotes in quotation marks "like this" to indicate direct quotes from your manuscript), or elaborate on this item by providing additional information not in the ms, or briefly explain why the item is not applicable/relevant for your study

"The source code of the POSTHOC app and SparkCare server are owned by Charles River Analytics and are confidential and proprietary under NIH SBIR 75N91021C00019. Researchers who would like to replicate, build upon, or collaborate on research involving these systems are encouraged to reach out to Charles River Analytics (www.cra.com). "

5-vii) Access

Access: Describe how participants accessed the application, in what setting/context, if they had to pay (or were paid) or not, whether they had to be a member of specific group. If known, describe how participants obtained “access to the platform and Internet” [1]. To ensure access for editors/reviewers/readers, consider to provide a “backdoor” login account or demo mode for reviewers/readers to explore the application (also important for archiving purposes, see vi).

	1	2	3	4	5	
subitem not at all important	○	○	◉	○	○	essential

Clear selection

Does your paper address subitem 5-vii? *

Copy and paste relevant sections from the manuscript (include quotes in quotation marks "like this" to indicate direct quotes from your manuscript), or elaborate on this item by providing additional information not in the ms, or briefly explain why the item is not applicable/relevant for your study

"The app and clinician portal are available through Charles River Analytic’s SparkCare platform. The app is freely downloadable from the Apple Store or Google Play. Participants need a personalized invitation from someone on our research team to set up an account in SparkCare and access the POSTHOC app."
We are happy to invite other beta-testers. Please email the corresponding author.

5-viii) Mode of delivery, features/functionalities/components of the intervention and comparator, and the theoretical framework

Describe mode of delivery, features/functionalities/components of the intervention and comparator, and the theoretical framework [6] used to design them (instructional strategy [1], behaviour change techniques, persuasive features, etc., see e.g., [7, 8] for terminology). This includes an in-depth description of the content (including where it is coming from and who developed it) [1]," whether [and how] it is tailored to individual circumstances and allows users to track their progress and receive feedback" [6]. This also includes a description of communication delivery channels and – if computer-mediated communication is a component – whether communication was synchronous or asynchronous [6]. It also includes information on presentation strategies [1], including page design principles, average amount of text on pages, presence of hyperlinks to other resources, etc. [1].

	1	2	3	4	5	
subitem not at all important	○	○	○	○	◉	essential

Clear selection

Does your paper address subitem 5-viii? *

Copy and paste relevant sections from the manuscript (include quotes in quotation marks "like this" to indicate direct quotes from your manuscript), or elaborate on this item by providing additional information not in the ms, or briefly explain why the item is not applicable/relevant for your study

A trained member of the study team will enter data from the medical record into the SCP in the app (Fig. 2). The interventionalists will be trained and will use a checklist to ensure fidelity and prevent drift. The participant will have a one-on-one meeting with an interventionalist and choose whether they want to prioritize nutrition or exercise as part of the study.

Table 1. Comparison of tools available to the intervention and control groups through the POSTHOC app

Feature	Intervention	Control
Receive Survivorship Care Plan (SCP)	✓ (in the app)	✓ (on paper)
Ability to enter key symptoms during Ecological Momentary Assessment	✓	✓
Access to Fitbit	✓	✓
Access to diet tracking feature	✓	-
Access to comparison between their steps, minutes of moderate-vigorous exercise, dietary intake, and sleep time compared to the SCP guidelines	✓	-
Location to log daily symptoms in the app	✓	-

Participants who choose to focus on exercise will be encouraged but not required to use their activity tracker during weeks 1-5 and 7-11 so that they can monitor their activity against their goals more easily. Over the course of the 12-week intervention, patients will gradually build up to the American College of Sports Medicine (ACSM) Physical Activity Guidelines for Cancer Survivors [30]. They will start at their baseline steps level and increase their daily step goal by 5-10% each week (towards 10,000 steps per day, if possible). They will aim to complete 20-30 minutes of moderate intensity aerobic exercise (40-59% heart rate reserve) per day, for a total of at least 150 minutes a week. They will also be encouraged to undergo stretching and resistance training of all major muscle groups two times a week, when possible.

Participants who choose to focus on nutrition will be encouraged to answer brief questions at the end of each day regarding their intake of fruits, vegetables, whole grains, and sweets to compare their eating habits to their goals in the POSTHOC app on a daily basis (See Supplementary Materials Table S1). Entry is possible for previous days if they forget to log. Goals will be based on the SCP guidelines, including working towards or maintaining a healthy BMI and consuming at least five servings of fruits and vegetables per day. Their goal will be to increase their adherence score for their nutrition goals, which will be determined through their questionnaire responses and the dietary assessment tool.

5-ix) Describe use parameters

Describe use parameters (e.g., intended “doses” and optimal timing for use). Clarify what instructions or recommendations were given to the user, e.g., regarding timing, frequency, heaviness of use, if any, or was the intervention used ad libitum.

	1	2	3	4	5	
subitem not at all important	○	○	○	◉	○	essential

Clear selection

Does your paper address subitem 5-ix?

Copy and paste relevant sections from the manuscript (include quotes in quotation marks "like this" to indicate direct quotes from your manuscript), or elaborate on this item by providing additional information not in the ms, or briefly explain why the item is not applicable/relevant for your study

"Participants who choose to focus on exercise will be encouraged but not required to use their activity tracker during weeks 1-5 and 7-11 so that they can monitor their daily activity against their goals more easily. "

"Participants who choose to focus on nutrition will be encouraged to answer brief questions at the end of each day regarding their intake of fruits, vegetables, whole grains, and sweets to compare their eating habits to their goals in the POSTHOC app on a daily basis "

5-x) Clarify the level of human involvement

Clarify the level of human involvement (care providers or health professionals, also technical assistance) in the e-intervention or as co-intervention (detail number and expertise of professionals involved, if any, as well as “type of assistance offered, the timing and frequency of the support, how it is initiated, and the medium by which the assistance is delivered”. It may be necessary to distinguish between the level of human involvement required for the trial, and the level of human involvement required for a routine application outside of a RCT setting (discuss under item 21 – generalizability).

	1	2	3	4	5	
subitem not at all important	○	○	○	○	●	essential

Clear selection

Does your paper address subitem 5-x?

Copy and paste relevant sections from the manuscript (include quotes in quotation marks "like this" to indicate direct quotes from your manuscript), or elaborate on this item by providing additional information not in the ms, or briefly explain why the item is not applicable/relevant for your study

"While the Survivorship Care Plan will be manually entered into the POSTHOC app for this trial, it is an ultimate goal for the platform to interface with the medical record so that the plan can be updated as a person’s health and goals change. "

5-xi) Report any prompts/reminders used

Report any prompts/reminders used: Clarify if there were prompts (letters, emails, phone calls, SMS) to use the application, what triggered them, frequency etc. It may be necessary to distinguish between the level of prompts/reminders required for the trial, and the level of prompts/reminders for a routine application outside of a RCT setting (discuss under item 21 – generalizability).

	1	2	3	4	5	
subitem not at all important	○	●	○	○	○	essential

Clear selection

Does your paper address subitem 5-xi? *

Copy and paste relevant sections from the manuscript (include quotes in quotation marks "like this" to indicate direct quotes from your manuscript), or elaborate on this item by providing additional information not in the ms, or briefly explain why the item is not applicable/relevant for your study

"EMA will be assessed via the POSTHOC app four times throughout the day for seven days at weeks 0, 6, and 12. The app will alert the participant to report the severity of four common sy4mptoms—pain, distress, fatigue, numbness/tingling, and interference of symptoms with daily activity on a scale of 0-10. While research studies have shown success with this type of data collection [32], participants will have the option to “turn off notifications” if it is too burdensome. The POSTHOC app does not have any other notifications other than the EMAs. "

5-xii) Describe any co-interventions (incl. training/support)

Describe any co-interventions (incl. training/support): Clearly state any interventions that are provided in addition to the targeted eHealth intervention, as ehealth intervention may not be designed as stand-alone intervention. This includes training sessions and support [1]. It may be necessary to distinguish between the level of training required for the trial, and the level of training for a routine application outside of a RCT setting (discuss under item 21 – generalizability.

	1	2	3	4	5	
subitem not at all important	◉	○	○	○	○	essential

Clear selection

Does your paper address subitem 5-xii? *

Copy and paste relevant sections from the manuscript (include quotes in quotation marks "like this" to indicate direct quotes from your manuscript), or elaborate on this item by providing additional information not in the ms, or briefly explain why the item is not applicable/relevant for your study

N/A - no co-interventions

6a) Completely defined pre-specified primary and secondary outcome measures, including how and when they were assessed

Does your paper address CONSORT subitem 6a? *

Copy and paste relevant sections from the manuscript (include quotes in quotation marks "like this" to indicate direct quotes from your manuscript), or elaborate on this item by providing additional information not in the ms, or briefly explain why the item is not applicable/relevant for your study

"The primary aim is to assess the feasibility, acceptability, and usability of the POSTHOC app by cancer survivors (n=36 randomized to the app group). The secondary aim is to obtain a preliminary estimate of the effect of the POSTHOC app vs. usual care on cumulative patient-reported symptom burden (n=54, 36 in the intervention arm and 18 in the usual care arm). The tertiary aim is to obtain a preliminary estimate of the effect of the POSTHOC app vs. usual care on patient-reported and objectively measured health behaviors (physical activity, diet, sleep). Exploratory mechanistic aims are to 1) assess cross-sectional associations between health behaviors (physical activity, diet, sleep) and cumulative symptom burden at baseline, 2) explore associations between changes in health behaviors (physical activity, diet, sleep) and cumulative symptom burden from baseline to 6 weeks and baseline to 12 weeks, and 3) explore associations between adherence to the intervention and changes in the same measures."

6a-i) Online questionnaires: describe if they were validated for online use and apply CHERRIES items to describe how the questionnaires were designed/deployed

If outcomes were obtained through online questionnaires, describe if they were validated for online use and apply CHERRIES items to describe how the questionnaires were designed/deployed [9].

	1	2	3	4	5	
subitem not at all important	◉	○	○	○	○	essential

Clear selection

Does your paper address subitem 6a-i?

Copy and paste relevant sections from manuscript text

Not applicable--we are administering validated, reliable questionnaires and our field does not distinguish between paper-based or computer-completed questionnaires.

6a-ii) Describe whether and how “use” (including intensity of use/dosage) was defined/measured/monitored

Describe whether and how “use” (including intensity of use/dosage) was defined/measured/monitored (logins, logfile analysis, etc.). Use/adoption metrics are important process outcomes that should be reported in any ehealth trial.

	1	2	3	4	5	
subitem not at all important	○	○	○	○	◉	essential

Clear selection

Does your paper address subitem 6a-ii?

Copy and paste relevant sections from manuscript text

"The primary outcome is the feasibility of the POSTHOC app, which will be determined by calculating the percentage of cancer patients in the POSTHOC group that used the app at least 3 times per week. We hypothesize that at least 75% of patients (≥ 27/36) will view or log data in the app at least 3 times per week at weeks 6 and 12. "

6a-iii) Describe whether, how, and when qualitative feedback from participants was obtained

Describe whether, how, and when qualitative feedback from participants was obtained (e.g., through emails, feedback forms, interviews, focus groups).

	1	2	3	4	5	
subitem not at all important	○	○	○	◉	○	essential

Clear selection

Does your paper address subitem 6a-iii?

Copy and paste relevant sections from manuscript text

"After completion of the study (or earlier if a participant withdraws early from the study), a member of the study team will conduct an exit interview to understand the participants' experience of being in the study. We will ask them if they think the app was helpful for alleviating their symptoms and improving their health behaviors. We will ask them what they liked about the study, did not like about it, what features they liked/did not like about the POSTHOC app (for those in the intervention arm), if they would recommend the study to others, etc. In order to get more information regarding patient-provider communication, we will ask about the number and quality of conversations between a patient and their provider(s) regarding physical activity and diet, as well as questions to understand patients' preferences and expectations in regard to where (e.g., clinic, home), when, how often, and with whom (e.g., primary care physician, oncologist) patient-provider communication should take place regarding lifestyle behaviors. This semi-structured interview will use qualitative methods approaches and will last approximately 30 minutes [51]. It will be audio-recorded with explicit permission at the time of the interview, then transcribed for qualitative and mixed methods data analysis. "

6b) Any changes to trial outcomes after the trial commenced, with reasons

Does your paper address CONSORT subitem 6b? *

Copy and paste relevant sections from the manuscript (include quotes in quotation marks "like this" to indicate direct quotes from your manuscript), or elaborate on this item by providing additional information not in the ms, or briefly explain why the item is not applicable/relevant for your study

No changes have been made to the trial outcomes.

7a) How sample size was determined

NPT: When applicable, details of whether and how the clustering by care provides or centers was addressed

7a-i) Describe whether and how expected attrition was taken into account when calculating the sample size

Describe whether and how expected attrition was taken into account when calculating the sample size.

	1	2	3	4	5	
subitem not at all important	○	○	○	○	●	essential

Clear selection

Does your paper address subitem 7a-i?

Copy and paste relevant sections from manuscript title (include quotes in quotation marks "like this" to indicate direct quotes from your manuscript), or elaborate on this item by providing additional information not in the ms, or briefly explain why the item is not applicable/relevant for your study

"Power calculations were based on data from a prior single-arm pilot study that we conducted among a similar group of cancer survivors (NIH project no. 75N91019C00026). Results from this analysis of covariance (ANCOVA) analysis suggested a pre-post correlation of 0.65 [52]. With two-tailed α=0.15 [46], a sample size of n=46 (after 15% missing data and 2:1 ratio of sample in arms) would provide 80% power to detect a standardized between-arm difference of 0.50, which corresponds to a medium-sized and clinically meaningful effect [53]. Preliminary data generated from another previous study of ours among cancer survivors [54] suggested a standard deviation of cumulative symptom burden from a similar symptom inventory of 17.0. Based on the estimated effect size for this study—0.50—we expect that the average cumulative symptom burden will decrease 8.5 points more in the POSTHOC group compared to the control group from baseline to 12 weeks (about 3 months)."

7b) When applicable, explanation of any interim analyses and stopping guidelines

Does your paper address CONSORT subitem 7b? *

Copy and paste relevant sections from the manuscript (include quotes in quotation marks "like this" to indicate direct quotes from your manuscript), or elaborate on this item by providing additional information not in the ms, or briefly explain why the item is not applicable/relevant for your study

No interim analyses are planned. No stopping guidelines are in place.

8a) Method used to generate the random allocation sequence

NPT: When applicable, how care providers were allocated to each trial group

Does your paper address CONSORT subitem 8a? *

Copy and paste relevant sections from the manuscript (include quotes in quotation marks "like this" to indicate direct quotes from your manuscript), or elaborate on this item by providing additional information not in the ms, or briefly explain why the item is not applicable/relevant for your study

"We will use a computer-generated table with random blocks of 3 and 6 and stratify by whether the participant had chemotherapy or not (yes/no), because symptoms and side effects of chemotherapy vs. radiation tend to differ. The randomization table was generated by the statistician (SZ) and is stored in the REDCap database; allocation is concealed from study staff who will be recruiting and randomizing the participants. "

8b) Type of randomisation; details of any restriction (such as blocking and block size)

Does your paper address CONSORT subitem 8b? *

Copy and paste relevant sections from the manuscript (include quotes in quotation marks "like this" to indicate direct quotes from your manuscript), or elaborate on this item by providing additional information not in the ms, or briefly explain why the item is not applicable/relevant for your study

"We will use a computer-generated table with random blocks of 3 and 6 and stratify by whether the participant had chemotherapy or not (yes/no)..."

9) Mechanism used to implement the random allocation sequence (such as sequentially numbered containers), describing any steps taken to conceal the sequence until interventions were assigned

Does your paper address CONSORT subitem 9? *

Copy and paste relevant sections from the manuscript (include quotes in quotation marks "like this" to indicate direct quotes from your manuscript), or elaborate on this item by providing additional information not in the ms, or briefly explain why the item is not applicable/relevant for your study

"The randomization table was generated by the statistician (SZ) and is stored in the REDCap database; allocation is concealed from study staff who will be recruiting and randomizing the participants. "

10) Who generated the random allocation sequence, who enrolled participants, and who assigned participants to interventions

Does your paper address CONSORT subitem 10? *

Copy and paste relevant sections from the manuscript (include quotes in quotation marks "like this" to indicate direct quotes from your manuscript), or elaborate on this item by providing additional information not in the ms, or briefly explain why the item is not applicable/relevant for your study

"The randomization table was generated by the statistician (SZ) and is stored in the REDCap database; allocation is concealed from study staff who will be recruiting and randomizing the participants. "

11a) If done, who was blinded after assignment to interventions (for example, participants, care providers, those assessing outcomes) and how

NPT: Whether or not administering co-interventions were blinded to group assignment

11a-i) Specify who was blinded, and who wasn’t

Specify who was blinded, and who wasn’t. Usually, in web-based trials it is not possible to blind the participants [1, 3] (this should be clearly acknowledged), but it may be possible to blind outcome assessors, those doing data analysis or those administering co-interventions (if any).

	1	2	3	4	5	
subitem not at all important	○	○	○	○	◉	essential

Clear selection

Does your paper address subitem 11a-i? *

Copy and paste relevant sections from the manuscript (include quotes in quotation marks "like this" to indicate direct quotes from your manuscript), or elaborate on this item by providing additional information not in the ms, or briefly explain why the item is not applicable/relevant for your study

"They will be randomized 2:1, POSTHOC:usual care (unblinded). "
"The statistician cannot be blinded due to the 2:1 randomization scheme."

11a-ii) Discuss e.g., whether participants knew which intervention was the “intervention of interest” and which one was the “comparator”

Informed consent procedures (4a-ii) can create biases and certain expectations - discuss e.g., whether participants knew which intervention was the “intervention of interest” and which one was the “comparator”.

	1	2	3	4	5	
subitem not at all important	○	○	○	○	◉	essential

Clear selection

Does your paper address subitem 11a-ii?

Copy and paste relevant sections from the manuscript (include quotes in quotation marks "like this" to indicate direct quotes from your manuscript), or elaborate on this item by providing additional information not in the ms, or briefly explain why the item is not applicable/relevant for your study

"They will be randomized 2:1, POSTHOC:usual care (unblinded). "

11b) If relevant, description of the similarity of interventions

(this item is usually not relevant for ehealth trials as it refers to similarity of a placebo or sham intervention to a active medication/intervention)

Does your paper address CONSORT subitem 11b? *

Copy and paste relevant sections from the manuscript (include quotes in quotation marks "like this" to indicate direct quotes from your manuscript), or elaborate on this item by providing additional information not in the ms, or briefly explain why the item is not applicable/relevant for your study

"To our knowledge, four SCP mobile applications (apps) are under development [20-24], addressing requests from patients, clinicians, and organizations. First, Baseman et al. [20] evaluated the feasibility and acceptability of SmartSurvivor, a mobile breast cancer survivorship care app in a pilot study. The goals of the app were to facilitate care coordination; support medical, psychosocial, and practical needs; and improve survivors' long-term health outcomes [20]. Overall, both survivors and providers felt that SmartSurvivor was a potentially valuable tool to support long-term SCP objectives [20]. Second, Denzen et al. [22] and Preussler et al. [21] are designing an online survivorship program for recipients of hematopoietic cell transplants called INSPIRE, which includes a mobile app. In focus groups, patients appreciated the comprehensiveness of the information, noting that some material was not provided by their providers [21]. They also noted its usefulness in guiding communication with physicians. However, they would have liked to see more personalized information (e.g., on transplant-induced gastrointestinal upset) as well as a means to monitor and track certain aspects of health [21]. Third, Murphy et al. [23] are designing a mobile app to deliver the SCP to survivors of childhood cancer. The authors previously tested a portable, credit card-sized passport to improve the survivors' knowledge of diagnosis, treatment, risks, and follow-up care. Patients who received the passport demonstrated improved and sustained knowledge compared to those who did not receive the passport until more than four months later [23]. The authors are following up on these results to develop a survivorship mobile phone app and suggest further study on the strengths and weakness of a mobile app approach [23]. Fourth, Stan et al. [24] are designing an app-based, electronic health record-integrated interactive care plan for breast cancer survivors. The goals of the app are to make the survivorship care plan into an actionable and engaging guide for patients and provide remote monitoring services for primary care providers [24]. The implementation of the app with 23 patients was feasible and created minimal provider burden, but patient engagement was below the feasibility threshold, suggesting that changes that may enhance adoption [24]. There are other digital and mobile applications designed to aid cancer survivors with self-management, but they do not deliver the SCP [25, 26]. "

12a) Statistical methods used to compare groups for primary and secondary outcomes

NPT: When applicable, details of whether and how the clustering by care providers or centers was addressed

Does your paper address CONSORT subitem 12a? *

Copy and paste relevant sections from the manuscript (include quotes in quotation marks "like this" to indicate direct quotes from your manuscript), or elaborate on this item by providing additional information not in the ms, or briefly explain why the item is not applicable/relevant for your study

"Statistical analysis for aims
Aim 1 assesses feasibility, acceptability, and usability. For feasibility, we will calculate the percentage of patients who used the POSTHOC app at least 3 times per week, as explained above. For acceptability, at weeks 6 and 12, we will estimate the percent of patients who: 1) found the app useful (≥4 on a scale of 1-7), 2) would recommend it to others (≥4 on a scale of 1-7), and 3) would use the app in the future (at least monthly). For usability, we will calculate summary statistics based on an overall usability score from the SUS survey (score range 0-100) [49]. A score of 68 ± 12.5 is considered average with >68 points “above average,’ and <68 “below average” [50]; these values are relevant for digital health apps [55]. We will review the qualitative data within the acceptability/usability surveys and during interviews to look for general themes related to specific design features that participants liked or that make the app challenging to use or understand.

Aim 2 assesses the effects of the intervention vs. control on cumulative symptom burden. Our data from Aim 2 will provide estimated effect sizes to inform the sample size of a confirmatory phase III randomized controlled trial, per clinical trial guidelines [56]. Cumulative symptom burden will be calculated as the sum of the individual severity levels from the 13 core symptoms from the MDASI (total symptom burden ranging 0-130) [34, 35]. We will use linear mixed modeling with total symptom burden at Week 6 as the primary symptom outcome, study arm (POSTHOC app vs. usual care) as the main independent variable, and total symptom burden at Week 0 as a covariate. In the case of significant imbalance in other factors between arms at baseline (e.g., age), we might include additional covariates in the model. We chose Week 6 as the primary time point because preliminary data from our group shows greater benefits of behavioral interventions earlier into behavioral interventions rather than later (Kleckner IR et al., under review and [57]). We will use the intent-to-treat principle and include each participant in the arm to which they were assigned regardless of use of the app for those assigned to the POSTHOC arm. We will also assess the effects of the intervention vs. usual care at week 12 as an exploratory aim.

Aim 3 will evaluate the effects of the intervention on Similar linear mixed models will evaluate the effects of the POSTHOC app vs. usual care for other outcomes at Week 6 and Week 12. To evaluate the longitudinal trajectory of each outcome, we will use repeated measures linear mixed models incorporating the specific outcome at all three time points and using a linear contrast to test effects of arm.

Exploratory analyses will include test of mediation and moderation effects on the POSTHOC app by physical activity adherence, sex, age, etc. [58-60]."

12a-i) Imputation techniques to deal with attrition / missing values

Imputation techniques to deal with attrition / missing values: Not all participants will use the intervention/comparator as intended and attrition is typically high in ehealth trials. Specify how participants who did not use the application or dropped out from the trial were treated in the statistical analysis (a complete case analysis is strongly discouraged, and simple imputation techniques such as LOCF may also be problematic [4]).

	1	2	3	4	5	
subitem not at all important	○	○	○	◉	○	essential

Clear selection

Does your paper address subitem 12a-i? *

Copy and paste relevant sections from the manuscript (include quotes in quotation marks "like this" to indicate direct quotes from your manuscript), or elaborate on this item by providing additional information not in the ms, or briefly explain why the item is not applicable/relevant for your study

"We will facilitate completion of all measures. The reasons for missing data will be tabulated according to treatment group (via interview, if possible) and participants who dropped out will be compared with those completed with regard to demographic and disease characteristics. The mechanism of missing data will be assessed to select appropriate statistical method (e.g., maximum likelihood weighting, multiple imputations)."

12b) Methods for additional analyses, such as subgroup analyses and adjusted analyses

Does your paper address CONSORT subitem 12b? *

Copy and paste relevant sections from the manuscript (include quotes in quotation marks "like this" to indicate direct quotes from your manuscript), or elaborate on this item by providing additional information not in the ms, or briefly explain why the item is not applicable/relevant for your study

"Exploratory analyses will include test of mediation and moderation effects on the POSTHOC app by physical activity adherence, sex, age, etc. [58-60]."

X26) REB/IRB Approval and Ethical Considerations [recommended as subheading under "Methods"] (not a CONSORT item)

X26-i) Comment on ethics committee approval

	1	2	3	4	5	
subitem not at all important	○	○	○	○	◉	essential

Clear selection

Does your paper address subitem X26-i?

Copy and paste relevant sections from the manuscript (include quotes in quotation marks "like this" to indicate direct quotes from your manuscript), or elaborate on this item by providing additional information not in the ms, or briefly explain why the item is not applicable/relevant for your study

"Before recruitment, approval was obtained by UMGCCC Clinical Research Center (CRC), UMSON, the University of Maryland Baltimore (UMB) Institutional Review Board (IRB; HP-00100473), and each of community oncology practices. "

x26-ii) Outline informed consent procedures

Outline informed consent procedures e.g., if consent was obtained offline or online (how? Checkbox, etc.?), and what information was provided (see 4a-ii). See [6] for some items to be included in informed consent documents.

	1	2	3	4	5	
subitem not at all important	○	○	◉	○	○	essential

Clear selection

Does your paper address subitem X26-ii?

Copy and paste relevant sections from the manuscript (include quotes in quotation marks "like this" to indicate direct quotes from your manuscript), or elaborate on this item by providing additional information not in the ms, or briefly explain why the item is not applicable/relevant for your study

"Participants will have the option of providing consent in-person using the computer (eConsent). For the written consent, a member of the study team will meet with the potential participant in a private room. She or he will go through the consent form with the patient face-to-face and answer any questions they may have. They will then be given the option to sign the consent form then or at a later date. Instead of the paper-based consent document, consent may occur using the IRB-approved eConsent document provided via REDCap. In this case, the study team reviews the consent form thoroughly via phone or videoconference (if they haven't already) to ensure comprehension. Once the eConsent form is signed and submitted, the patient will be able to receive a printout of the paper copy, download a digital version, and/or receive an email with a digital attachment of the signed form.
We will ensure that participants are aware that consent is a voluntary process and that they can withdraw at any time. Because this study includes only one "phase," we will not ask participants to re-consent during the study unless there is a change to the study procedures or other pertinent information that would change a person's decision to participate in the study. The consent form will also contain information regarding HIPAA authorization."

X26-iii) Safety and security procedures

Safety and security procedures, incl. privacy considerations, and any steps taken to reduce the likelihood or detection of harm (e.g., education and training, availability of a hotline)

	1	2	3	4	5	
subitem not at all important	○	○	●	○	○	essential

Clear selection

Does your paper address subitem X26-iii?

Copy and paste relevant sections from the manuscript (include quotes in quotation marks "like this" to indicate direct quotes from your manuscript), or elaborate on this item by providing additional information not in the ms, or briefly explain why the item is not applicable/relevant for your study

"Responses to questionnaires (e.g., On Study), as well as the Clinical Record Form, will be collected via REDCap, and therefore will be stored on UMB servers. Charles River Analytics will not have access to any of the data collected in this study."

"Because the SCP includes protected health information (PHI), we are ensuring the safety of the data using several layers of security. First, the SCP includes minimal PHI that could be used in a security breach; it does not include name, birthday, address, phone number, email address, or medical record number. Second, SparkCare software (Charles River Analytics, Cambridge, MA), which houses the POSTHOC app and the PostgreSQL database, is installed on UMB servers managed using Azure services (Microsoft Corp., Redmond, WA) so data are not transferred to or from Charles River Analytics or anywhere other than the participants' personal smartphones. Third, we installed an Internet Information Services (IIS) webserver; obtained a public IP address, domain name, and security certificate; and configured a domain name system (DNS). The POSTHOC app is not publicly available and can only be accessed via an internal developer link that is invitation only (via Test Flight for Apple users). Google and Fitbit accounts are made specifically for this study instead of any personal Google account the participant might already have.
The POSTHOC app also has a "clinician portal" on a dual authentication-protected website. The clinician portal allows study team members to manage participants, assign participants to the POSTHOC or usual care arm, and enter the SCP. Only the study team has access to the study data, though the participant may share the data with their provider at their discretion."

"Participants' privacy and confidentiality will be protected in several ways. At the beginning of a session, each participant will be assigned an alphanumeric code that will be written on all their response materials (except for their consent form) and data files instead of their names. This procedure will ensure that participants' identities will never be directly associated with their specific data. To protect participant confidentiality, all paperwork that could directly be linked to the participant will be stored in the investigators' locked filing cabinets. All other data will be coded (i.e., identified by code rather than with personal information) and stored in a locked cabinet or on a computer where a password is required for entry and decryption. The Site PI, Dr. Kleckner, will distribute passwords and keys. Only adequately trained study staff who require data access to complete the study will receive a password or key.

Privacy will be ensured by making appointments with participants to call them or have a video conference about the study. If a participant elects to come in, our clinical suites are located in a discrete laboratory in the School of Nursing."

RESULTS

13a) For each group, the numbers of participants who were randomly assigned, received intended treatment, and were analysed for the primary outcome

NPT: The number of care providers or centers performing the intervention in each group and the number of patients treated by each care provider in each center

Does your paper address CONSORT subitem 13a? *

Copy and paste relevant sections from the manuscript (include quotes in quotation marks "like this" to indicate direct quotes from your manuscript), or elaborate on this item by providing additional information not in the ms, or briefly explain why the item is not applicable/relevant for your study

N/A This is a protocol paper

13b) For each group, losses and exclusions after randomisation, together with reasons

Does your paper address CONSORT subitem 13b? (NOTE: Preferably, this is shown in a CONSORT flow diagram) *

Copy and paste relevant sections from the manuscript (include quotes in quotation marks "like this" to indicate direct quotes from your manuscript), or elaborate on this item by providing additional information not in the ms, or briefly explain why the item is not applicable/relevant for your study

N/A This is a protocol paper

13b-i) Attrition diagram

Strongly recommended: An attrition diagram (e.g., proportion of participants still logging in or using the intervention/comparator in each group plotted over time, similar to a survival curve) or other figures or tables demonstrating usage/dose/engagement.

	1	2	3	4	5	
subitem not at all important	(●)	( )	( )	( )	( )	essential

Clear selection

Does your paper address subitem 13b-i?

Copy and paste relevant sections from the manuscript or cite the figure number if applicable (include quotes in quotation marks "like this" to indicate direct quotes from your manuscript), or elaborate on this item by providing additional information not in the ms, or briefly explain why the item is not applicable/relevant for your study

N/A This is a protocol paper

14a) Dates defining the periods of recruitment and follow-up

Does your paper address CONSORT subitem 14a? *

Copy and paste relevant sections from the manuscript (include quotes in quotation marks "like this" to indicate direct quotes from your manuscript), or elaborate on this item by providing additional information not in the ms, or briefly explain why the item is not applicable/relevant for your study

N/A This is a protocol paper

14a-i) Indicate if critical “secular events” fell into the study period

Indicate if critical “secular events” fell into the study period, e.g., significant changes in Internet resources available or “changes in computer hardware or Internet delivery resources”

	1	2	3	4	5	
subitem not at all important	◉	○	○	○	○	essential

Clear selection

Does your paper address subitem 14a-i?

Copy and paste relevant sections from the manuscript (include quotes in quotation marks "like this" to indicate direct quotes from your manuscript), or elaborate on this item by providing additional information not in the ms, or briefly explain why the item is not applicable/relevant for your study

N/A This is a protocol paper

14b) Why the trial ended or was stopped (early)

Does your paper address CONSORT subitem 14b? *

Copy and paste relevant sections from the manuscript (include quotes in quotation marks "like this" to indicate direct quotes from your manuscript), or elaborate on this item by providing additional information not in the ms, or briefly explain why the item is not applicable/relevant for your study

N/A This is a protocol paper

15) A table showing baseline demographic and clinical characteristics for each group

NPT: When applicable, a description of care providers (case volume, qualification, expertise, etc.) and centers (volume) in each group

Does your paper address CONSORT subitem 15? *

Copy and paste relevant sections from the manuscript (include quotes in quotation marks "like this" to indicate direct quotes from your manuscript), or elaborate on this item by providing additional information not in the ms, or briefly explain why the item is not applicable/relevant for your study

N/A This is a protocol paper

15-i) Report demographics associated with digital divide issues

In ehealth trials it is particularly important to report demographics associated with digital divide issues, such as age, education, gender, social-economic status, computer/Internet/ehealth literacy of the participants, if known.

	1	2	3	4	5	
subitem not at all important	(•)	( )	( )	( )	( )	essential

Clear selection

Does your paper address subitem 15-i? *

Copy and paste relevant sections from the manuscript (include quotes in quotation marks "like this" to indicate direct quotes from your manuscript), or elaborate on this item by providing additional information not in the ms, or briefly explain why the item is not applicable/relevant for your study

N/A This is a protocol paper

16) For each group, number of participants (denominator) included in each analysis and whether the analysis was by original assigned groups

16-i) Report multiple “denominators” and provide definitions

Report multiple “denominators” and provide definitions: Report N’s (and effect sizes) “across a range of study participation [and use] thresholds” [1], e.g., N exposed, N consented, N used more than x times, N used more than y weeks, N participants “used” the intervention/comparator at specific pre-defined time points of interest (in absolute and relative numbers per group). Always clearly define “use” of the intervention.

	1	2	3	4	5	
subitem not at all important	(●)	( )	( )	( )	( )	essential

Clear selection

Does your paper address subitem 16-i? *

Copy and paste relevant sections from the manuscript (include quotes in quotation marks "like this" to indicate direct quotes from your manuscript), or elaborate on this item by providing additional information not in the ms, or briefly explain why the item is not applicable/relevant for your study

N/A This is a protocol paper

16-ii) Primary analysis should be intent-to-treat

Primary analysis should be intent-to-treat, secondary analyses could include comparing only “users”, with the appropriate caveats that this is no longer a randomized sample (see 18-i).

	1	2	3	4	5	
subitem not at all important	( )	( )	( )	(●)	( )	essential

Clear selection

Does your paper address subitem 16-ii?

Copy and paste relevant sections from the manuscript (include quotes in quotation marks "like this" to indicate direct quotes from your manuscript), or elaborate on this item by providing additional information not in the ms, or briefly explain why the item is not applicable/relevant for your study

"We will use the intent-to-treat principle and include each participant in the arm to which they were assigned regardless of use of the app for those assigned to the POSTHOC arm. "

17a) For each primary and secondary outcome, results for each group, and the estimated effect size and its precision (such as 95% confidence interval)

Does your paper address CONSORT subitem 17a? *

Copy and paste relevant sections from the manuscript (include quotes in quotation marks "like this" to indicate direct quotes from your manuscript), or elaborate on this item by providing additional information not in the ms, or briefly explain why the item is not applicable/relevant for your study

N/A This is a protocol paper.

17a-i) Presentation of process outcomes such as metrics of use and intensity of use

In addition to primary/secondary (clinical) outcomes, the presentation of process outcomes such as metrics of use and intensity of use (dose, exposure) and their operational definitions is critical. This does not only refer to metrics of attrition (13-b) (often a binary variable), but also to more continuous exposure metrics such as "average session length". These must be accompanied by a technical description how a metric like a "session" is defined (e.g., timeout after idle time) [1] (report under item 6a).

	1	2	3	4	5	
subitem not at all important	(●)	( )	( )	( )	( )	essential

Clear selection

Does your paper address subitem 17a-i?

Copy and paste relevant sections from the manuscript (include quotes in quotation marks "like this" to indicate direct quotes from your manuscript), or elaborate on this item by providing additional information not in the ms, or briefly explain why the item is not applicable/relevant for your study

N/A This is a protocol paper.

17b) For binary outcomes, presentation of both absolute and relative effect sizes is recommended

Does your paper address CONSORT subitem 17b? *

Copy and paste relevant sections from the manuscript (include quotes in quotation marks "like this" to indicate direct quotes from your manuscript), or elaborate on this item by providing additional information not in the ms, or briefly explain why the item is not applicable/relevant for your study

N/A This is a protocol paper.

18) Results of any other analyses performed, including subgroup analyses and adjusted analyses, distinguishing pre-specified from exploratory

Does your paper address CONSORT subitem 18? *

Copy and paste relevant sections from the manuscript (include quotes in quotation marks "like this" to indicate direct quotes from your manuscript), or elaborate on this item by providing additional information not in the ms, or briefly explain why the item is not applicable/relevant for your study

N/A This is a protocol paper.

18-i) Subgroup analysis of comparing only users

A subgroup analysis of comparing only users is not uncommon in ehealth trials, but if done, it must be stressed that this is a self-selected sample and no longer an unbiased sample from a randomized trial (see 16-iii).

	1	2	3	4	5	
subitem not at all important	(selected)	○	○	○	○	essential

Clear selection

Does your paper address subitem 18-i?

Copy and paste relevant sections from the manuscript (include quotes in quotation marks "like this" to indicate direct quotes from your manuscript), or elaborate on this item by providing additional information not in the ms, or briefly explain why the item is not applicable/relevant for your study

N/A This is a protocol paper.

19) All important harms or unintended effects in each group

(for specific guidance see CONSORT for harms)

Does your paper address CONSORT subitem 19? *

Copy and paste relevant sections from the manuscript (include quotes in quotation marks "like this" to indicate direct quotes from your manuscript), or elaborate on this item by providing additional information not in the ms, or briefly explain why the item is not applicable/relevant for your study

N/A This is a protocol paper.

19-i) Include privacy breaches, technical problems

Include privacy breaches, technical problems. This does not only include physical “harm” to participants, but also incidents such as perceived or real privacy breaches [1], technical problems, and other unexpected/unintended incidents. “Unintended effects” also includes unintended positive effects [2].

	1	2	3	4	5	
subitem not at all important	(selected)					essential

Clear selection

Does your paper address subitem 19-i?

Copy and paste relevant sections from the manuscript (include quotes in quotation marks "like this" to indicate direct quotes from your manuscript), or elaborate on this item by providing additional information not in the ms, or briefly explain why the item is not applicable/relevant for your study

N/A This is a protocol paper.

19-ii) Include qualitative feedback from participants or observations from staff/researchers

Include qualitative feedback from participants or observations from staff/researchers, if available, on strengths and shortcomings of the application, especially if they point to unintended/unexpected effects or uses. This includes (if available) reasons for why people did or did not use the application as intended by the developers.

	1	2	3	4	5	
subitem not at all important	(selected)					essential

Clear selection

Does your paper address subitem 19-ii?

Copy and paste relevant sections from the manuscript (include quotes in quotation marks "like this" to indicate direct quotes from your manuscript), or elaborate on this item by providing additional information not in the ms, or briefly explain why the item is not applicable/relevant for your study

N/A This is a protocol paper.

DISCUSSION

22) Interpretation consistent with results, balancing benefits and harms, and considering other relevant evidence

NPT: In addition, take into account the choice of the comparator, lack of or partial blinding, and unequal expertise of care providers or centers in each group

22-i) Restate study questions and summarize the answers suggested by the data, starting with primary outcomes and process outcomes (use)

Restate study questions and summarize the answers suggested by the data, starting with primary outcomes and process outcomes (use).

	1	2	3	4	5	
subitem not at all important	○	○	◉	○	○	essential

Clear selection

Does your paper address subitem 22-i? *

Copy and paste relevant sections from the manuscript (include quotes in quotation marks "like this" to indicate direct quotes from your manuscript), or elaborate on this item by providing additional information not in the ms, or briefly explain why the item is not applicable/relevant for your study

N/A This is a protocol paper.

But we do restate the questions:

22-ii) Highlight unanswered new questions, suggest future research

Highlight unanswered new questions, suggest future research.

	1	2	3	4	5	
subitem not at all important	○	○	◉	○	○	essential

Clear selection

Does your paper address subitem 22-ii?

Copy and paste relevant sections from the manuscript (include quotes in quotation marks "like this" to indicate direct quotes from your manuscript), or elaborate on this item by providing additional information not in the ms, or briefly explain why the item is not applicable/relevant for your study

N/A This is a protocol paper.

20) Trial limitations, addressing sources of potential bias, imprecision, and, if relevant, multiplicity of analyses

20-i) Typical limitations in ehealth trials

Typical limitations in ehealth trials: Participants in ehealth trials are rarely blinded. Ehealth trials often look at a multiplicity of outcomes, increasing risk for a Type I error. Discuss biases due to non-use of the intervention/usability issues, biases through informed consent procedures, unexpected events.

	1	2	3	4	5	
subitem not at all important	○	○	●	○	○	essential

Clear selection

Does your paper address subitem 20-i? *

Copy and paste relevant sections from the manuscript (include quotes in quotation marks "like this" to indicate direct quotes from your manuscript), or elaborate on this item by providing additional information not in the ms, or briefly explain why the item is not applicable/relevant for your study

"bias may be introduced into this study in that only people who are comfortable enough with technology to beta-test an app will enroll. Those who enroll and are less comfortable with technology might be more likely to remain in the study if they are in the control group vs. the POSTHOC group, but drop-out rates should not affect our primary outcome (feasibility, acceptability, and usability, which analyzes only data from the POSTHOC arm), or symptom data in Aim 2. While we might have POSTHOC participants who don't use the app (non-adherence), it will not be possible for control participants to use the app (contamination) because we have control over what features all the participants can see. Non-adherence will lead to more conservative effect size estimates of the effect of the intervention on symptom burden. "

21) Generalisability (external validity, applicability) of the trial findings

NPT: External validity of the trial findings according to the intervention, comparators, patients, and care providers or centers involved in the trial

21-i) Generalizability to other populations

Generalizability to other populations: In particular, discuss generalizability to a general Internet population, outside of a RCT setting, and general patient population, including applicability of the study results for other organizations

	1	2	3	4	5	
subitem not at all important	○	○	○	○	○	essential

Does your paper address subitem 21-i?

Copy and paste relevant sections from the manuscript (include quotes in quotation marks "like this" to indicate direct quotes from your manuscript), or elaborate on this item by providing additional information not in the ms, or briefly explain why the item is not applicable/relevant for your study

Your answer

21-ii) Discuss if there were elements in the RCT that would be different in a routine application setting

Discuss if there were elements in the RCT that would be different in a routine application setting (e.g., prompts/reminders, more human involvement, training sessions or other co-interventions) and what impact the omission of these elements could have on use, adoption, or outcomes if the intervention is applied outside of a RCT setting.

	1	2	3	4	5	
subitem not at all important	○	●	○	○	○	essential

Clear selection

Does your paper address subitem 21-ii?

Copy and paste relevant sections from the manuscript (include quotes in quotation marks "like this" to indicate direct quotes from your manuscript), or elaborate on this item by providing additional information not in the ms, or briefly explain why the item is not applicable/relevant for your study

"It is the ultimate goal for the POSTHOC app to be available to anyone with an Apple or Android smartphone. While the features of the app would remain the same between real-world and research settings, there would be one main difference in the set-up: the research team is currently manually entering the SCP into the app for the participant. In the real-world, the clinical care team should not be burdened with entering the SCP into a separate application, but the ultimate goal would be for app to interface with the electronic medical record so that the app automatically has the SCP and continuously updates as the user's health status and goals change. "

OTHER INFORMATION

23) Registration number and name of trial registry

Does your paper address CONSORT subitem 23? *

Copy and paste relevant sections from the manuscript (include quotes in quotation marks "like this" to indicate direct quotes from your manuscript), or elaborate on this item by providing additional information not in the ms, or briefly explain why the item is not applicable/relevant for your study

"Registration: ClinicalTrials.gov NCT05499663;
https://clinicaltrials.gov/ct2/show/NCT05499663 "

24) Where the full trial protocol can be accessed, if available

Does your paper address CONSORT subitem 24? *

Cite a Multimedia Appendix, other reference, or copy and paste relevant sections from the manuscript (include quotes in quotation marks "like this" to indicate direct quotes from your manuscript), or elaborate on this item by providing additional information not in the ms, or briefly explain why the item is not applicable/relevant for your study

This is the full trial protocol :)

25) Sources of funding and other support (such as supply of drugs), role of funders

Does your paper address CONSORT subitem 25? *

Copy and paste relevant sections from the manuscript (include quotes in quotation marks "like this" to indicate direct quotes from your manuscript), or elaborate on this item by providing additional information not in the ms, or briefly explain why the item is not applicable/relevant for your study

Your answer

X27) Conflicts of Interest (not a CONSORT item)

X27-i) State the relation of the study team towards the system being evaluated

In addition to the usual declaration of interests (financial or otherwise), also state the relation of the study team towards the system being evaluated, i.e., state if the authors/evaluators are distinct from or identical with the developers/sponsors of the intervention.

	1	2	3	4	5	
subitem not at all important	○	○	○	◉	○	essential

Clear selection

Does your paper address subitem X27-i?

Copy and paste relevant sections from the manuscript (include quotes in quotation marks "like this" to indicate direct quotes from your manuscript), or elaborate on this item by providing additional information not in the ms, or briefly explain why the item is not applicable/relevant for your study

"ATW and NMM are employed by Charles River Analytics. Other authors have no conflicts of interest."

About the CONSORT EHEALTH checklist

As a result of using this checklist, did you make changes in your manuscript? *

- ( ) yes, major changes
- (•) yes, minor changes
- ( ) no

What were the most important changes you made as a result of using this checklist?

We added more details to the methods regarding recruitment, what

How much time did you spend on going through the checklist INCLUDING making changes in your manuscript *

Your answer

As a result of using this checklist, do you think your manuscript has improved? *

- (x) yes
- ( ) no
- ( ) Other:

Would you like to become involved in the CONSORT EHEALTH group?

This would involve for example becoming involved in participating in a workshop and writing an "Explanation and Elaboration" document

- ( ) yes
- (x) no
- ( ) Other:

Clear selection

Any other comments or questions on CONSORT EHEALTH

Your answer

STOP - Save this form as PDF before you click submit

To generate a record that you filled in this form, we recommend to generate a PDF of this page (on a Mac, simply select "print" and then select "print as PDF") before you submit it.

When you submit your (revised) paper to JMIR, please upload the PDF as supplementary file.

Don't worry if some text in the textboxes is cut off, as we still have the complete information in our database. Thank you!

Final step: Click submit !

Click submit so we have your answers in our database!

Submit

Clear form

Never submit passwords through Google Forms.

This content is neither created nor endorsed by Google. Report Abuse - Terms of Service - Privacy Policy

Google Forms
