# Supplementary material for: Digitizing Survivorship Care Plans Through the POST-Treatment Health Outcomes of Cancer Survivors (POSTHOC) Mobile App: Protocol for a Phase II Randomized Controlled Trial
Source: JMIR Res Protoc. 2024 Sep 5;13:e59222. doi: 10.2196/59222 (PMC11413545; doi:10.2196/59222)
Supplement: Multimedia Appendix 4 [file resprot_v13i1e59222_app4.pdf]

Technical Questions
1. *While a particular strength of the proposal in terms of design and rigor, there is a question as to the feasibility of conducting the 3-month (per participant) RCT in the second year. Please address.*

We selected the three-month duration, as suggested by Co-I, Dr. Shayne, as the clinically relevant time point after completion of cancer treatment, as that is typically when patients are seen for a follow-up visit. We agree with the reviewer that this may be a long period for a survivor to evaluate our tool. Our initial proposal conservatively planned on the three-month period, however, we will assess primary intervention outcomes at six weeks, whereas the outcomes at three months are merely exploratory. Six weeks is short enough period of evaluation based on our prior experience, while long enough to capture meaningful effects of health behaviors (e.g., exercise) on patient symptoms.

*2. Relevant behavioral science and technological frameworks are noted and consistently mentioned but not thoroughly translated into clear driving elements of the proposed work. Please address.*

We thank the reviewers for the question. We have added additional details to the best of our ability to infer the intent of the question. We would be happy to provide further details, if needed. To aid our description, we included the system architecture from our original proposal (Figure 1).

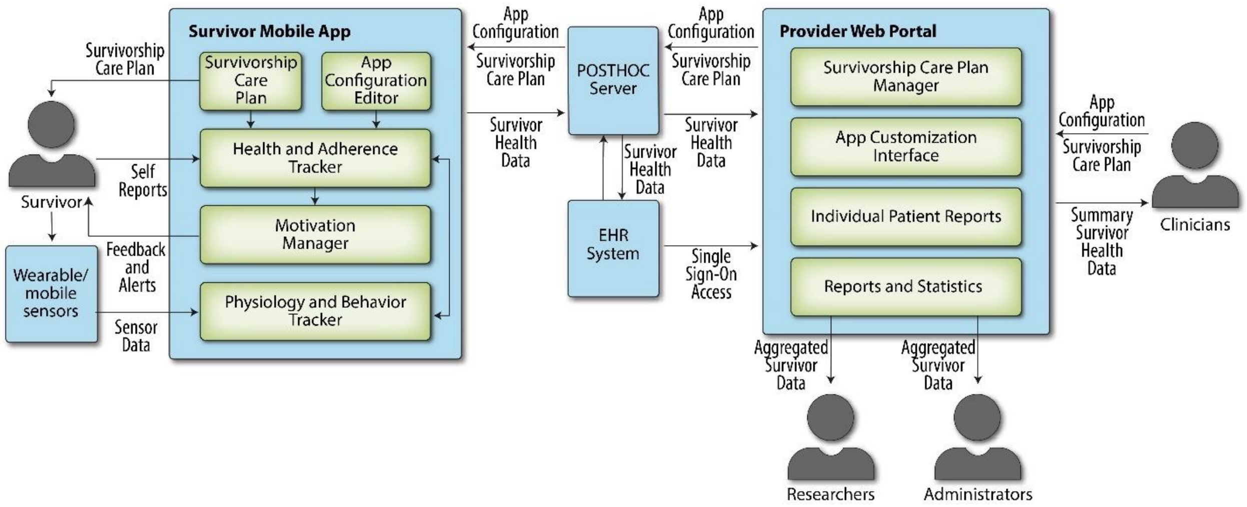


*Figure 1: POSTHOC system architecture*

The central goal of the POSTHOC proposal is the creation, maintenance, and adherence to a digital survivorship care plan (SCP). The principal driving technical components to meet the goal of Phase II POSTHOC is the *Survivor Mobile App* and the *Provider Web Portal*. The Survivor Mobile App provides the patient-facing resources that reinforce the SCP beyond the walls of the clinic by collecting health data and providing feedback and resources to promote health management. The Provider Web Portal enables clinical care team members to monitor survivor health and adherence to SCPs, facilitate communication with the survivor, and enable inquiry about the health data. Essentially, the central vision for POSTHOC is to translate SCPs into a digital "workbook" that guides daily behavior and informs the patient/provider relationship. We acknowledge that SCPs have fallen somewhat out of favor since our Phase I effort. However, we believe that the intent of SCPs toward tracking symptoms, ensuring good daily health behaviors, and facilitating communication between survivors and providers about health goals are key to good survivorship care.

From our Phase I requirements analysis, we identified four main barriers to effective survivorship care. First, we learned that a main frustration from a clinical perspective is that the SCPs are time consuming to create and are considered "dead" once the document is passed off to the survivor, meaning that the survivor takes the information with them but it does not lead to any further action. This appears to arise from the format of the SCP (i.e., a static paper document) and the difficulty of coordinating action among the care team (e.g., oncologists, primary care providers; PCPs) which places the impetus on the survivor to pass information about cancer care among the care team. Furthermore, the SCP rarely gets updated as the health of the survivor evolves. SCPs typically have general guidelines and symptoms, but these lists are rarely updated according to the survivors' ongoing health outcomes. We determined that using the *Provider Web Portal* to create a digital SCP that can be

iteratively updated as health evolves and is readily available to survivors and healthcare teams would go a long way to mitigating this challenge.

Second, interviewees frequently mentioned that patients were disconcerted with the lack of a care coordination in the survivorship phase after having had such an involved, and often large, group of care providers invested in their treatment. This appears to be exacerbated by the effort required of providers to dig up the SCP document, if it is available at all. Furthermore, there is little information on the SCP that designates which provider is responsible for each element of the plan. These issues require providers to consistently review and research the survivors' history as their health evolves. Another frustration expressed by SCP researchers and PCPs is the difficulty of identifying survivors from the electronic health record (EHR). PCPs must review the details of the patient's chart to find the history of cancer and chemotherapy. We determined that the *Provider Web Portal* could integrate into the EHR via the Epic App Orchard marketplace and indicate that a patient is a cancer survivor, providing access from within the patients' chart that can connect them directly to the SCP and act as a hub to coordinate post-treatment cancer care.

Third, the current approach to an SCP does not tap into the wealth of available data through mobile, wearable, and connected devices. Clinicians and researchers agreed that the SCP should function as a jumping off point for a set of tools that assist in implementing the health guidelines. An SCP should encompass the health history and guidelines but should also be regularly updated and compared to regular data from the survivor. The *Survivor Mobile App* provides ready access to the digital SCP created by the provider in the *Provider Web Portal* and facilitates the configuration of wearable and smart devices to reinforce the recommendations and guidelines of the SCP. The integration of the SCP onto a mobile platform and collection of relevant health data provides valuable longitudinal insights about the behavior and state of the survivor between visits and are not subjective to bias from recall. They can also be used to provide an at-a-glance summary to the survivor and the care team indicating the progress against SCP guidelines.

Finally, our analysis revealed that the ability to complete objective health assessments outside the clinic would be a great help in ongoing cancer care, especially as the population ages and deals with comorbidities that impact a patients ability to meet key post-treatment goals (e.g., arthritis negatively impact ability to exercise). The complicated overlap among conditions over time can lead to many visits to the clinic and the investment of substantial hospital resources. At-home assessments can provide a first line of investigation to prioritize and triage the daily symptoms and health concerns that can occur daily. The *Survivor Mobile App* collects patient reported outcomes and providing data about clinical endpoints and minimally important differences (MID) to allow healthcare teams to tap into the most useful and actionable information that the platform can provide.

We hope that this distills the driving motivation for the POSTHOC platform. Additional details about other elements mentioned in the proposal, such as the behavioral science principals to ensure that digital SCPs effectively reinforce behavior change, the privacy and security architecture and procedures to ensure that digital SCPs and corresponding health information is not compromised, and integration with EHRs to ensure that digital SCPs dovetail with existing clinical workflows, are provided in the proposal and we would be happy to provide additional details about these supporting components. We have also included Table 1 to provide an at-a-glance view of these central themes.

*Table 1: Summary of POSTHOC technical components requirements to address key barriers in SCP care*

| Barrier | POSTHOC Requirements to Address Barrier |
|---|---|
| SCPs should provide tools to help the survivor manage health, but usually become a "dead" document after they are delivered | Care Team Members (CTMs) must be able to create and update a digital version of the SCP along with health guidelines and recommendations:<br>▪ Must be able to define survivor's goals and set methods to track them |
| | The digital SCP must be easily available to the survivor and should be updated as their health evolves:<br>▪ Must be able to connect to patients between appointments<br>▪ Must be able to track adherence to guidelines and prescriptions (e.g., medication adherence)<br>▪ Must be able to gather/monitor information about survivor behavioral goals<br>▪ Must be able to gather/monitor information about likely symptoms and long-term effects<br>▪ Must be able to gather/monitor information about well-being<br>▪ Must incorporate behavioral science informed mechanisms to drive engagement<br>▪ Must have instructions for how to read and use POSTHOC and SCPs |

| | |
|---|---|
| | CTMs and survivors must be able to configure the behavior of mobile tools (e.g., smartphones, watches, Internet of Things (IoT) devices) to reinforce the SCP:<br>▪ Must be able to connect survivors and their devices with available resources (e.g., current apps and tools, community resources)<br>▪ Must be able to set reminders for follow-up appointments, labs, and screenings |
| Effective care coordination is difficult among the members of the care team (e.g., Oncologists, PCP) | The tool should flag the electronic medical records of survivors to indicate to non-oncologists that the digital SCP exists |
| | The tool should provide fast access to the digital SCP |
| | When in the tool, CTMs and survivors should have a quick reference of who is on the care team |
| | When in the tool, CTMs and survivors should have an effective means of recording and passing information:<br>▪ Must be able to contact other members of the care team |
| | The digital SCP should clearly designate the CTM responsible for follow up on all items of the plan |
| | The tool should assist CTMs in identifying the follow up items under their purview |
| | Where possible, the tool should use current tools (e.g., MyChart, eRecord, InBasket) to reduce the user burden |
| Care teams are missing out on key sources of information about health behaviors that are often already collected through wearables and smartphone apps (e.g., Apple Watch, Fitbit, food diaries) | The tool should provide an informative at-a-glance summary to providers of SCP adherence and health status:<br>▪ Must be able to access reports on the progress status of survivors (e.g., overdue for a follow up?)<br>▪ Must also be able alert care team related to urgent needs (e.g., indications of a desire for self-harm)<br>▪ Must have access to contextual data when possible to aid in interpretation of tracked/reported exercise (e.g., comparison to group or individual baseline) |
| | The tool should assist survivors in reviewing their progress against the guidelines of the SCP |
| | The tool should leverage existing technology as much as possible to reduce the learning curve and increase usage |
| There is limited means to gather objective, valid clinical endpoints outside the clinic | The tool should be designed with clinical endpoints, and minimal important differences (MID) in mind to give CTMs direct access to actionable data. |
| | The tool should be designed to minimize the burden of integrating new wearable and at-home diagnostic devices into the platform. |

*3. No discussion of the evidence of the efficacy of the few existing mHealth apps for childhood cancers discussed that would inform the refinement of the current app proposed in this proposal. Please address.*

This question has been rescinded by the NCI Contracting Officer.

*4. The limited inclusion of actual cancer survivors who will be using the apps is concerning. In Phase 1 only a single survivor was involved. The proposal correctly states that the success of the platform will be in its reliability, ease of use and accessibility. A major concern is the low involvement of survivors. Please address.*

We are glad that the reviewers agree with us that acceptance of the tool by cancer survivors is important. During the Phase I effort, we planned to run the usability study proposed in Section 2.4.5.1. However, the pandemic disrupted these plans. Rather than extend our Phase I effort, we opted to replace the usability study with virtual outreach to clinicians and researchers and move the usability studies to the early stages of Phase II.

We will interact with cancer survivors at three stages during the Phase II effort. The first is in our formative usability evaluations with cancer survivors (9 months after contract; MAC). During this phase of the effort, we will interact with 5-7 cancer survivors who will be asked to perform several tasks with the Phase II prototype and speak aloud their thoughts. The key observations for our team during this phase will be to identify potential points of error, confusion, frustration, and other preferences. While these sample sizes seem small for research in medicine and healthcare, we want to emphasize that the purpose of this usability study to identify design issues specific to UIs rather than to make any generalizable conclusions about healthcare or treatment. Additionally, the literature on the topic of formative usability tests indicate that the majority of the critical or unique design issues can be identified with these sample sizes (Nielsen, 1993).

We also believe that it is crucial to achieve buy in for the product from user groups (as the reviewers point out in Question 9. The University of Rochester has access to several Survivorship groups (e.g., Clinical Translational Science Institute (CTSI), Cancer and Aging Research Group (CARG)) that will allow us to solicit

information feedback about the desirability and usefulness of the POSTHOC platform from potential users. Between 9-12 MAC, we will respond to feedback from the first usability study and iteratively demonstrate the tool to these survivorship groups to gather additional feedback and begin to win buy in from survivors who would gain benefit from digital SCPs.

After gathering the formative feedback from Cancer Survivors, we will return to our prototype development to make refinements before conducting another formal usability study with Cancer Survivors. During this study, we will ask the Survivors recruited during our clinical randomized control trial (RCT; N=54) to participate in a lifestyle alignment study. During this study, survivors will be given a packet of tasks to be evaluated at home in a self-moderated study. The packet will also include one or more surveys about the feasibility, usability, and design of the POSTHOC platform that should be completed periodically. Finally, we will use the Phase I/II feasibility/preliminary efficacy RCT to gather further information about the feasibility, acceptability, and usability of the app by patients, by monitoring usage patterns. These metrics will be in addition to the aim of obtaining a preliminary assessment of the effect of the POSTHOC app.

Based on these plans, we estimate that we will be able to gather information from >60 survivors about various dimensions of the POSTHOC platform.

*5. A major weakness of the current plan is the lack of detail around survivor involvement. In the proposed model, rather than allowing the targeted survivors to use a prototype app and provide feedback, the approach focuses on running focus-group type sessions. Details of how many users will be included, at what point of the development, and exactly how their feedback will be incorporated is unclear. The design choices may be supported by scientific literature, but whether following these principles will lead to a system that is beneficial to the patients depends on how they feel about using it. Please address these concerns.*

As mentioned in our response to Question 4, our solicitation of feedback from users will have several phases that range from an initial focus group-style usability study (see Section 2.4.5.1), to a lifestyle alignment study which examines at home usage patterns and feedback (see Section 2.4.5.2), to an evaluation within a clinical environment (see Section 2.5.4.3). We believe that these activities involving survivors will provide a solid foundation for planning iterative prototype development cycles and improving the Phase II POSTHOC platform.

6. *The robust platform could be overwhelming in its scope—might there be ways to prioritize or constrain active elements to preferred elements and tracking (some assessments appear adaptable). Please address.*

We thank the reviewers for this insightful observation and agree that "scope-creep" is undesirable. There are many directions and benefits that POSTHOC can provide as a platform, many of which are mentioned in our proposal to communicate our long-term vision. We hope we provided a more distilled picture of the central elements to POSTHOC in our response to Question 2. In essence, the Phase II effort of POSTHOC will focus on effective creation, management, and facilitation of adherence to digital SCPs. During the Phase II effort, we will work closely with our partners at URMC to ensure that we first prioritize the assessments and features that will improve the chances of success in our clinical evaluation. As part of our efforts to transition and commercialize POSTHOC, we will be in near-constant outreach to researchers and clinicians at other institutions who are involved in survivorship care (e.g., we are already providing updates to researchers and providers at the University of Vermont, Fred Hutch, University of Washington, and University of Cincinnati about our progress). As opportunities arise to transition POSTHOC into use at other institutions, we will prioritize planned features that increase the likelihood of commercial success.

*7. It is difficult to determine whether the end product or technology proposed would offer significant advantages over existing approaches, methodologies, instrumentation, or interventions currently utilized in research or clinical practice for commercial application. Due to the dearth of available mHealth apps for CCS, any new protocol type creates the opportunity to provide a technical innovation for clinical practice. Please address.*

This question has been rescinded by the NIH Contracting Officer.

8. *The potential advantages of the UI over existing UIs from the perspective of innovation is unclear. Please address.*

There are several components that will require UI development. First, the *Survivor Mobile App,* will have UIs that allow survivors to reference their digital SCP and track their progress against clinician recommendations/guidelines. To our knowledge, there are no existing UIs for mobile display of a digital SCP. Although a digital SCP will be new on mobile devices, we determined that Survivors will be most likely to use UIs that they are familiar with. Thus, the UI designs shown in Section 2.4.2 of the proposal are based on the UIs already present on the mobile platform. For example, the wireframe in Figure 9 of the proposal is based on the UI conventions for the Apple Health App. This will provide the advantage of familiar visual cues and functions to navigate through the app.

The second component requiring UI development is the *Provider Web Portal*. Under Phase I, we created wireframe designs for several key features needed in the web portal. From our user requirements analysis and past experience designing tools for health care providers, we created a view that gives a bottom line up front (BLUF) view of the onboarding status of the survivorship care plan and a view of the adherence results (see Figure 14 in the proposal). Based on feedback from our subject matter experts, we designed the Provider Portal to intelligently adapt its display elements and functionality to the care team role of a given provider. For example, if the user is in an administrative role, we would adapt the BLUF view to focus on the onboarding status and adherence to follow-ups/screenings/labs while deemphasizing other details. The user can access additional details on the BLUF view items by clicking on the "plus" icons next to each item. This populates information and controls related to the SCP (see Figure 15 in the proposal), the configuration of the POSTHOC mobile app (see Figure 16 in the proposal), and adherence to SCP guidelines (see Figure 17, 18 in the proposal). The conceptual advantage of these design elements is to provide easily accessible, targeted, information to busy clinical teams with additional details on demand. In our Phase I design concepts, we also created wireframes for a UI that allows the creation and configuration of the digital SCP. These concepts rely heavily on "drag and drop" UI features and sliders. This is to address the feedback from our Phase I requirements analysis that creating an SCP is a very time-consuming activity. During Phase II, we will be continually working with partners at URMC and our commercialization targets to get feedback on these UI elements to ensure that they are easy to use and effective.

*9. One potential omission from the commercialization equation involves substantial engagement with payers and how they would respond to this platform. Insurers are briefly mentioned, but this could be expanded and support a more compelling argument to other potential partners and licensees. Please address.*

Thanks to the reviewers for this comment. We plan to develop plans for commercialization through medical billing / charge codes after POSTHOC has matured as a platform. In the near term, our plan is to commercialize POSTHOC through direct sales and partnerships with researchers and clinicians in cancer care. We have already had success with this model in the commercialization of our CAMPFIRE effort, which aims for initial market capture and brining in funds for further development. The CAMPFIRE platform is being piloted at the Kansas University Medical Center, and the University of Iowa and being considered for inclusion in R01 grants which will provide additional funds for continuing development and maintaining the platform. Meanwhile our outreach to clinicians has created a direct licensing opportunity with the University of Cincinnati, who are offering to license our platform in 4-5 of their clinics. We plan to offer CAMPFIRE to them on a yearly license which will provide the funds to continue development and maintain the platform.

This will be the model for our near term commercialization efforts. As POSTHOC is able to capture customers through academic partnerships (including mentions in academic publications) and direct sales, we will begin to refine our model by supporting the platform through reimbursement through medical billing.

*10. While distinct stakeholders are considered, at the end of the day, the entire mechanism will only work if there is a community of survivors willing to use the software. Perhaps greater involvement of survivors in the first two phases would improve chances of commercial success. Please address.*

This is a very insightful comment. We would add that buy in is important from provider groups as well since they will be ultimately making the decision to adopt the tool in their clinic, creating the digital SCPs, and helping survivors monitor adherence to the guidelines. To achieve buy in from the providers, we will be conducting regular demonstrations with researchers and clinicians involved in survivorship care. We have already received interest from staff at the University of Vermont, Fred Hutch, University of Washington, and University of Cincinnati about our progress. We plan to continue and expand this outreach to gather feedback about the

usefulness of POSTHOC and its features, and keep potential users apprised of our progress. These clinicians and researchers will be our first commercial targets. Under our related CAMPFIRE effort (a cognitive assessment platform for CRCI), we have had good success marketing our tool as a research and clinical tool. Researchers have begun adopting CAMPFIRE in their R01 research efforts, providing funds to our group for feature development and technical support. Clinicians have been in talks with our group about direct licensing of the tool for use in their clinics.

Regarding buy in from survivors, we agree! As mentioned in our response to Question 4, we plan to conduct survivor outreach to demonstrate the tool, gather high level feedback, and achieve buy in between 6-9 MAC. We will focus on gathering feedback from survivor groups at our partner institution (e.g., CTSI and CARG) to get access to Breast and Colorectal cancer populations.

*11. It was not evident that a team member has experience as a care provider or a researcher of AYA CCS. Please address.*

This question has been rescinded by the NIH contracting officer.

*12. Can you please advise the status of your IRB approval?*

We will obtain IRB approval for the formative and lifestyle alignment usability studies early in the first year of our effort. We will work with URMC to obtain institutional approval, including IRB clearance, throughout the first year of the effort.

*13. Please confirm understanding that any resultant award will be a restricted award (no human subjects research can occur) unless and until IRB approval is obtained.*

Confirmed.

*14. Please confirm whether this project involves surveys or other data collection involving 10 or more respondents. If so, additional OMB clearances or information may be required when data is collected from 10 or more individuals. If required, I will facilitate any clearance needed.*

We plan to gather information from more than 10 respondents and will work with the Sponsor to gather OMB clearance.

*15. Please confirm the extent to which any Personally Identifiable Information (PII) and/or any private, sensitive information will be collected from participants (e.g., medical or health information, educational information, financial information). Please describe the variables or data points, and provide a list of research questions if available, regarding these sensitive and/or private topics. If PII will be collected, is that information housed in a system designed to retrieve that PII by a unique identifier (i.e., name, SSN, patient ID number, etc.). In other words, an identifier that is linked to only one individual.*

Preserving the privacy of Protected Healthcare Information (PHI) is critical and required by law. Preservation of PHI requires a significant investment in our corporate infrastructure, with implications affecting the policies and procedures related to areas such as our information technology (IT) and data systems, human resources and personnel training, and legal obligations. Given that healthcare is a strategic area for Charles River, we are currently in the process of drafting and implementing these policies and procedures to become compliant with HIPAA regulations. We anticipate a successful HIPAA compliance audit through HITRUST to be completed by 31 March 2021. Mr. Geoff Catto and Mr. Austin Crumpton are members of the Charles River HIPAA Task Force and are entrusted with the responsibility to ensure that our company and our POSTHOC effort will be able to protect PHI and other data gathered, processed, and/or stored in the course of our Phase II development effort and beyond. We will also develop POSTHOC so that licensees or customers will be able to integrate a HIPAA compliant version of POSTHOC with their own IT systems. Charles River Analytics adheres to several other Federal compliance laws, including the DFARS 252.204-7012 clause which mandates the NIST 800-171 guide

to protect Controlled Unclassified Information and all contractor information systems. We also have a security program and adhere to the NIST 800-53 guide for protecting classified information.

The data transmission protocol must also ensure that PHI is not compromised, and that falsified information cannot be pushed to the Provider Web Portal. To ensure privacy, all data not in transit over a network link will be stored on encrypted disks or kept behind the hospital's firewall (e.g., within the EHR database). Furthermore, we will implement transport layer security (TLS) for data encryption between the mobile application and back-end server, which requires mobile apps to be authorized before connection to the server endpoint. This secure data transfer approach supports three types of communication:

1. **Mobile application pulling information from the server.** The pull function occurs when the user requests data from the server. Each time this occurs, a map is created to a server endpoint and data is returned in an open data-exchange format. If the request has come from an authenticated source, the framework will be authorized to pull the requested data from the server and push it to the mobile application. The fidelity of this transfer will be achieved by a handshake mechanism, which ensures the reliability of messages received, provides delivery guarantees, and avoids message duplication.
2. **Mobile application pushing information to the server.** The push function works in a similar way to the pulling of information, except that the request to pull information also includes a data payload. One challenge of pushing information to a server is intermittent network connectivity, which can be overcome through a network-aware message queue. This framework ensures that messages successfully reach the server endpoint even if delivery is delayed until connectivity is re-established.
3. **Server pushing information to the mobile application.** To receive reminders, messages, and alerts from the Provider Web Portal, the mobile application must also be capable of passively receiving data. Mobile operating systems (OS) only allow apps to pull data from a server when the app is opened, however, the capability of apps to receive push-notifications on mobile OS will allow the Provider Web Portal to push information to the mobile app on demand. Once the app is opened, the pull function can be executed as described in item 1 above or additional routines can be enabled to receive the required data.

*16. Please complete an updated Protection of Human Subjects form, attached.*

See attached.

*17. Please describe how the subcontractor and/or consultant will be involved in recruitment and/or data collection. Any organization/individual participating in Human Subjects research must be covered under an active FWA. It is noted that consultants, unlike subcontractors, are not typically directly involved in recruitment and/or data collection activities. Please address.*

Our team will recruit 54 patients with cancer through clinics of Co-I, Dr. Michelle Shayne, and colleagues (see Letter of Support in Section **Error! Reference source not found.** of the proposal). The recruitment and data collection activities will be the primary task of the URMC team, which will allow tighter control over PHI (see Question 15). Recruitment will be feasible over two years given that URMC's Wilmot Cancer Institute completes treatment on over 200 patients per month who go on to receive a SCP, and these types of behavioral interventions typically consent 20% of approached patients (i.e., 3-5 per month for 10-15 months). Given the large >20,000 square mile catchment area (over 3 million people) of the Wilmot Cancer Center, we will also be able to recruit patients from sites outside Rochester, NY including patients who live in rural areas (37% of Wilmot's catchment population).

Randomization will occur 2:1 for POSTHOC app (N=36) versus control (N=18) will use a computer-generated table with random blocks of 3 and 6. We will stratify by whether the patient was just completing chemotherapy or just completing radiotherapy.

All assessments will occur at Week 0 (before randomization), Week 6 (mid-intervention), and Week 12 (post-intervention), except for (Aim 1) feasibility and acceptability and (Exploratory aim) healthcare utilization because they do not apply to Week 0. This study does not require any face-to-face contact for assessments, and can utilize either face-to-face or telehealth meetings for consent and to demonstrate the use of the activity tracker and the POSTHOC app.

To assess the feasibility and acceptability of the POSTHOC app, we will assess the frequency by which both patients and clinicians view or log data in the POSTHOC app and how much patients and providers enjoy using

the POSTHOC app on a Likert scale from 1-5 (i.e., strong dislike, dislike, neither like nor dislike, like, strong like).

To evaluate the cumulative symptom burden, we will use the sum of severities of the core patient-reported symptoms from the MD Anderson Symptom Inventory including fatigue, insomnia, pain, appetite loss, dyspnea, cognitive problems, anxiety, nausea, depression, sensory neuropathy, constipation, and diarrhea. The patient will rate the severity of each symptom from 0 (symptom not present) to 10 (symptom as bad as you can imagine) in the past week.

To gather patient-reported and objectively measured health behaviors (physical activity, diet, sleep), we will configure the POSTHOC platform to target patient-reported measures, physical activity (GODIN Physical Activity Questionnaire, daily steps, daily minutes of moderate-vigorous physical activity), diet (NCI Fruit & Vegetable screener), smoking (have you smoked in the past six weeks, average number of uses in the past six weeks) including any tobacco or vaping products, alcohol consumption (average number of alcoholic drinks in the past six weeks), and sleep quality (Insomnia Severity Index). We will also report multi-question scales of key symptoms, including neuropathy (CIPN-206), pain (BPI7), cognitive impairment (FACT-Cog8), fatigue (Brief Fatigue Inventory; BFI9), anxiety and depression (HADS10 and Distress Thermometer11), and sleep disturbances (ISI5), clinician-reported patient health (e.g., ECOG performance status), and healthcare utilization (e.g., number of unplanned medical encounters).

During the RCT, URMC researchers will access medical record information including age, sex, race, ethnicity, height, weight, infusions date, dose, delays, other medications, and co-morbidities (e.g., hypertension, diabetes) to control for confounding variables and make specific consideration of sex and other biological variables in our reports.

Our data will more accurately estimate effect sizes to inform the sample size of an R01 for a confirmatory Phase III RCT, per clinical trial guidelines (Freidlin et al., 2012). Hypotheses will be tested at $\alpha=0.15$ (2-tailed) as appropriate for Phase I/II studies of this size (Rubinstein et al., 2005). To obtain a preliminary estimate of the effect of the POSTHOC app versus usual care on cumulative patient-reported symptom burden (via the MDASI1). The outcome is the cumulative severity of the core symptoms by adding the individual severity levels from the thirteen core symptoms of the MDASI. We will use the analysis of covariance (ANCOVA) with cumulative severity at Week 6 as an outcome, the study arm (exercise versus nutrition education control) as main independent variable, and CIPN at Week 0 as a covariate. Under assumptions of ANCOVA and pre-post correlation of 0.65 (per Dr. Kleckner's pilot study) (Borm et al., 2007), sample size N=50 (after 15% data loss) provides 80% power to detect a standardized between-arm difference of 0.50, which corresponds to a medium-sized and clinically meaningful effect (Norman et al., 2003).

Similar ANCOVA models will evaluate the effects of the POSTHOC app versus usual care each outcome at week six and at week twelve. To evaluate the longitudinal trajectory of each outcome, we will use repeated measures linear mixed model (LMM) (Kenward & Roger, 1997) incorporating the specific outcome at all three time points and using a linear contrast to test effects of POSTHOC app versus control.
